# Exploring the impact of gender-related variables on health measures and perceived stress

**DOI:** 10.3389/fpsyg.2025.1500674

**Published:** 2025-02-25

**Authors:** Maria Picó-Pérez, Marisa S. Coelho, Rita Vieira, Mafalda Machado-Sousa, Pedro Morgado

**Affiliations:** 1 Life and Health Sciences Research Institute (ICVS), University of Minho, Braga, Portugal; 2 ICVS/3B’s, PT Government Associate Laboratory, Braga, Portugal; 3 Departamento de Psicología Básica, Clínica y Psicobiología, Universitat Jaume I, Castellón de la Plana, Spain; 4 Clinical Academic Center - Braga, Braga, Portugal

**Keywords:** gender, stress, mental health, discrimination, social support

## Abstract

**Introduction:**

Despite its relevance for health outcomes, only recently gender has begun to be explored in the scientific literature, typically using a rigid binary framework. To tackle this, the Stanford Gender-related Variables for Health Research (GVHR) was developed to analyze gender from a multidimensional perspective. We aim to use our Portuguese version of the GVHR and analyze its association with health outcomes, including perceived stress.

**Methods:**

To this aim, 351 participants completed the GVHR scale, sociodemographic, and health information (including the Perceived Stress Scale, PSS-10). A Confirmatory Factor Analyses (CFA) was first performed, and logistic and linear regressions were used to explore the association between gender and health-related variables.

**Results:**

All measures of CFA showed appropriate goodness of fit. Regarding regression models, gender discrimination and higher levels of risk-taking were associated with binge drinking. Lower social support and risk-taking, and being male, were associated with being overweight. Regarding stress, it was positively associated with discrimination and work strain, while it was negatively associated with social support, emotional intelligence and risk-taking. Finally, discrimination and work strain were positively associated with mental health worsening and activity limitations, while social support was negatively associated with mental health worsening.

**Discussion:**

In conclusion, by approaching gender from a multidimensional perspective we detected specific factors influencing health outcomes, showing that the relational aspects of gender are particularly relevant for mental health.

## 1 Introduction

The exclusion of females from clinical trials as well as from many areas of basic research was a critical issue that harmed not only the health of women, but also the advance of science and medicine (Beery and Zucker, 2011). In order to correct this situation, the National Institutes of Health (NIH) and other funding agencies requested the inclusion of women in clinical trials (NIH Revitalization Act, 1993), and later the consideration of sex as a biological variable in basic research (Clayton and Collins, 2014). While these actions were necessary, they also led to an excessive focus on the search for sex differences, in many cases without previously reflecting on why sex was relevant for the research topic being studied, and on what was the best approach to explore its potential influence.

Importantly, several studies have shown that not only sex but also gender plays a determining role in health outcomes (Heise et al., 2019; Regitz-Zagrosek, 2012). In this line, the World Health Organization (WHO) assumed gender as one of the social determinants of population health and health inequalities in the Social Determinants of Health framework (Solar and Irwin, 2010). While sex generally refers to a set of biological attributes associated with physical and physiological features, gender refers to a multidimensional construct associated with social roles, behavior, lifestyle, and personal experiences (Heidari et al., 2016). Despite this differentiation, sex and gender are often inappropriately confused in the literature (Hammarström and Annandale, 2012), and gender is still rarely considered in health research, which could be due to the lack of quantitative tools to analyze its influence on health outcomes.

Moreover, even when they are explored, comparisons are usually made within a rigid binary framework, performing simple male vs. female analyses that leave sex and gender-related sources of variability without explanation (Joel, 2021; Sanchis-Segura and Wilcox, 2024). But individuals experience gender-related norms differently, and thus we miss information when we classify them as something with two opposite, fixed poles (Saguy et al., 2021). A previous gender scale that has been widely used, the Bem Sex Role Inventory (BSRI; Bem, 1974), challenges the assumption of masculinity and femininity being two opposed ends of the same dimension, but it still focusses solely in the psychological and individual aspects of gender roles, and is based on outdated notions of femininity and masculinity. While these concepts may be addressing some individual aspects of gender that are relevant for health (such as personality traits), there are other relational and institutional aspects of gender that may also be critical for our health (Connell, 2012; Heise et al., 2019). In this regard, measuring and labeling gender-related behaviors as such (e.g., caregiving or discrimination) could give richer information for individualized medicine than just sex or gender categories, or scores based on a single measure of masculinity/femininity based on individual traits.

Due to this gap in the field, a new instrument was developed for the North American population for the study of gender using a multidimensional approach - the Stanford Gender-related Variables for Health Research (GVHR), after a thorough review of the literature regarding gender dimensions and gender-related variables (Nielsen et al., 2021). Importantly, considering that gender is a social construct that can vary from one culture to another, it is necessary to analyze if gender measures created in one cultural group are valid for a different culture. For example, the GVHR scale was recently adapted to the Spanish population (Díaz-Morales et al., 2023), and the authors found that a five-factor model was more tenable for that population than the original seven-factor structure. Still, caregiver and work strain were the gender variables that predicted worse health-related quality of life, psychological health, and health-risk behaviors, in line with the findings from the original scale.

In the current work, we aim to translate and validate the Stanford GVHR instrument to the Portuguese population, and analyze the impact of gender-related variables on health outcomes. Moreover, we intend to go beyond the original article and explore the potential impact of gender-related variables on stress. According to the WHO, stress can be defined as a state of worry or mental tension caused by a difficult situation (World Health Organization, 2023). Stress is a natural human response that is experienced by everyone when facing challenges and threats in their lives. However, when prolonged, stress can become pathological, impacting a person’s physical and mental health (O’Connor et al., 2021). The impact of sex and gender on stress has been previously studied, using either a binary approach (Matud, 2004), or a dimensional approach but only considering physiological variables such as hormones (Kajantie and Phillips, 2006). Thus, exploring the impact of gender-related variables from a multidimensional perspective might help go one step beyond what is already known regarding sex/gender differences on stress.

## 2 Materials and methods

### 2.1 Ethical considerations

This project was conducted by the principles of the Declaration of Helsinki and was approved by the Ethical Subcommittee in Life and Health Sciences of the University of Minho (CEICVS 005/2022). Before completing the form, participants had to give their consent for data collection. All data was processed anonymously.

### 2.2 Scale translation

First, the original English version of the GVHR was obtained from the authors and translated to Portuguese by two Portuguese native speakers. Then, after a consensus was reached, an independent bilingual translator back-translated the scale, which was sent for approval to the original authors.

### 2.3 Data collection

This project had a cross-sectional design, and data collection took place between July 2022 and November 2022. The data was collected and managed using REDCap electronic data capture tools hosted at the researcher’s university (Harris et al., 2009, 2019). The questionnaire was shared through the university mailing lists with students, staff, and the general community. The time requested to complete the questionnaire was approximately 20 min. The only exclusion criteria applied was age less than 18 years or not giving consent to collect the data. The questionnaire was divided into four sections: social demographics information (16 questions), health information (10 questions), stress level information (Perceived Stress Scale – PSS-10), and gender-related variables information (GVHR).

Social demographics information included, among others, age, sex, gender, sexual orientation, relationship status, education, last year’s income, and professional status (for most variables we used the same questions and response options as in Nielsen et al., 2021).

The Health Days Measures (CDC HRQOL-4; Centers for Disease Control and Prevention, 2000) was used to collect health information, including general health status, physical health, mental health, and recent activity limitations. Additionally, weight, height, and questions about monthly habits, such as binge drinking, smoking, and vaping, were included in this section.

The Portuguese version of the PSS-10 (Trigo et al., 2010) was used to measure perceived stress. The PSS is a self-report measure designed to capture the degree to which situations in an individual’s life are appraised as stressful. It is composed of a five-point Likert scale varying from zero (never) to four (very often), corresponding to the frequency of stress felt in the last month. Scores range from 0 to 40, with higher scores corresponding to higher stress levels. In the validation of this scale by Trigo et al. (2010), it showed good reliability with a Cronbach’s alpha of 0.874.

Finally, the GVHR captures critical aspects of three dimensions of gender: Gender Norms (cultural rules produced through social institutions and cultural products), Gender-Related Traits (how individuals or groups perceive and present themselves concerning gender norms), and Gender Relations (how gender shapes social interactions in romantic relationships, friendships, families, schools, workplaces, and public settings). These are represented in the original validation of the questionnaire with seven factors: caregiver and work strain (Gender Norms), independence, risk-taking and emotional intelligence (Gender-Related Traits), and social support and discrimination (Gender Relations).

The complete instruments (in Portuguese) used for collecting the sociodemographic and health data, as well as the GVHR, can be found in the Supplementary material, while the original English version of these instruments can be found in the Supplementary material of Nielsen et al. (2021) publication.

### 2.4 Statistical analysis

Statistical analysis was performed using SPSS (Version 28, Chicago, IL, United States), Amos (Version 7.0) and JASP (Version 0.17.2, JASP Team, University of Amsterdam, the Netherlands). The significance level for all tests (*p*-value) was set at 0.05. The minimum sample size needed to validate the questionnaire was 260 participants, considering that the GVHR scale has 26 items, and there is a general recommendation of 10 participants per item (Boateng et al., 2018). Also, that sample size would allow us to detect effect sizes as small as *R*^2^ = 0.097 in multiple regression models (type-I error = 5%, statistical power = 95%, 10 predictors; calculated with GPower 3.1.9.2).

Before performing the statistical analysis, the database was appropriately prepared based on what was done in the original GVHR article. First, body mass index (BMI) was computed based on self-reported values on weight and height, and dichotomized for further analysis to reflect under or normal weight (BMI < 25 = 0) and overweight or obese (BMI ≥ 25 = 1). Current smoking and vaping, which were measured by the number of cigarettes smoked/vaped per day, were also dichotomized (not smoking = 0, smoking = 1; not vaping = 0, vaping = 1). Binge drinking was measured by the frequency of consuming five or more drinks on one occasion for males and four or more drinks on one occasion for females. We recoded these items into a unique dichotomic variable for all participants (binge drinking less than monthly = 0, binge drinking monthly, weekly, or daily/almost daily = 1). Finally, the general health variable was dichotomized into 0 for good, very good, or excellent responses and 1 for fair or poor.

**Figure 1:**
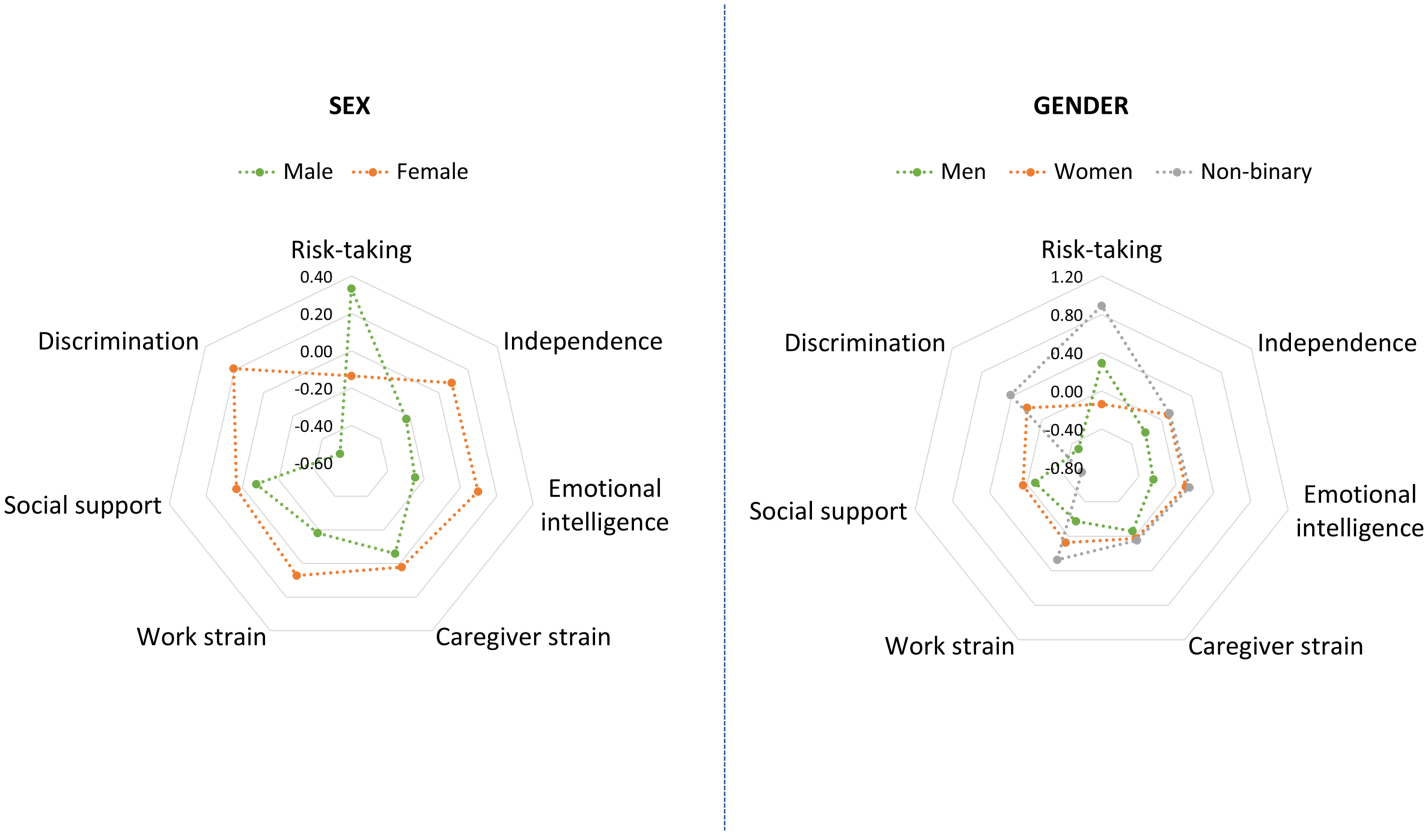
Mean z-scores for the seven GVHR factors, separately by sex (left) and by gender (right).

Regarding the GVHR questionnaire, we recoded the missing data into 1 for the variables caregiver strain and work strain, following the same approach as in the original study. That is, people not currently caring for someone in need or not currently employed (and thus not responding to the caregiver strain and work strain questions) were ascribed the value 1, which represents no strain due to caregiving/work. Finally, we calculated standardized z-scores for each variable from the GVHR questionnaire, and mean-item subscale scores were computed for each of the seven factors, to be used later as predictors in the regression analyses. All variables are scored from lower to higher levels of the given constructs. Figure 1 displays the mean z-scores for the seven GVHR factors, separately by sex as well as by gender.

A Confirmatory Factor Analysis (CFA) was performed using Amos (Version 7.0) and based on maximum likelihood estimations, to confirm the factor structure from the original scale. Model fit was based on several recommended criteria (Boateng et al., 2018), such as chi-square test (χ^2^), χ^2^/df ratio (2–5), comparative fit index (CFI > 0.90), goodness of fit index (GFI > 0.90), Tucker-Lewis index (TLI > 0.90) and root mean square error of approximation (RMSEA <0.05). Cronbach’s alpha coefficients were calculated for those measures composed by several items. Pearson correlation coefficients were also computed to analyze relationships between the seven GVHR factors.

Finally, we used JASP to explore the association between the seven factors and health-related variables. Logistic or linear regression was used depending on whether the dependent variable was categorical or continuous, respectively. Thus, logistic regression was used for the BMI, general health, vaping, smoking, and binge drinking dichotomized variables. On the other hand, linear regression was used for the PSS-10, physical health, mental health, and activity limitations. Moreover, age and years of education were included as covariates, in addition to either sex or gender (each model was performed twice), following what was done in the original publication.

## 3 Results

### 3.1 Sample characteristics

The sample included 351 participants (which was over the 260 needed), 100 male (28.5%), and 251 female (71.5%), with an average age of 30.4 (Min = 18 and Max = 64). Descriptive statistics on all the relevant sociodemographic and health variables are shown in Table 1. The Shapiro–Wilk test showed that the only continuous variable following a normal distribution was the PSS-10, so the mean and standard deviation (SD) are shown for this variable, while the median and interquartile range (IQR) are shown for the others.

**Table 1:** Sociodemographic and health characteristics of the sample.

Variables	Categories	*N*	%
Sex	Male	100	28.5
	Female	251	71.5
Gender	Men	102	29.1
	Women	244	69.5
	Non-binary	4	1.1
	Prefer not say	1	0.3
Sexual orientation	Exclusively heterosexual	224	63.8
	Mostly heterosexual	74	21.1
	Bisexual	21	6.0
	Mostly homosexual	7	2.0
	Exclusively homosexual	13	3.7
	Pansexual	2	0.6
	Asexual	8	2.3
	Prefer not to say	2	0.6
Relationship status	Single	250	71.2
	Married/living with a romantic partner	87	24.8
	Divorced/separated	13	3.7
	Widower	1	0.3
Education level	Basic school degree	3	0.9
	High school degree	90	25.6
	Bachelor’s degree	124	35.3
	Master’s degree	83	23.6
	Doctorate degree	51	14.5
Last year income	Less than 7000€	129	36.8
	7000€–10999€	31	8.8
	11000€–19999€	52	14.8
	20000€–24999€	27	7.7
	25000€–36999€	27	7.7
	37000€–79999€	22	6.3
	More than 80000€	3	0.9
	Prefer not to say	60	17.1
Professional status	Worker	148	42.2
	Student	193	55.0
	Unemployed	10	2.8
General health	Excellent, very good, good	312	88.9
	Fair, poor	39	11.1
Smoking	Yes	48	13.7
	No	303	86.3
Vaping	Yes	12	3.4
	No	339	96.6
Binge drinking*	Monthly, weekly, or daily	118	33.6
	Less than monthly	233	66.4
BMI	Under or normal weight (BMI < 25)	258	73.5
	Overweight or obese (BMI ≥ 25)	93	26.5
		Mean	SD
PSS-10		29.15	7.467
		Median	IQR
Age		25	16
Education years		17	4
Physical health		2	5
Mental health		8	16.5
Activity limitations		1	5

BMI, Body-mass index; SD, Standard Deviation; PSS-10, Perceived Stress Scale; IQR, Interquartile range. Physical/Mental health refer to the number of days with poor physical/mental health during the last 30 days, while Activity limitations refers to the number of days with activity limitations due to poor physical or mental health during the last 30 days.

*The naming and coding of this variable was maintained the same as in the original publication by Nielsen et al. (2021) for consistency, but it is important to note that only a 1.7% of the sample engaged in binge drinking daily or almost daily (with a 13.7% engaging weekly, and a 18.2% engaging monthly).

### 3.2 Confirmatory factor analysis

CFA of GVHR indicated acceptable fit, with χ^2^ = 456.058, χ^2^/df ratio = 1.788, CFI = 0.942, GFI = 0.903, TLI = 0.931, and RMSEA = 0.047. Also, all factors showed appropriate internal consistency (see Supplementary Table S1 for their Cronbach’s alpha coefficients), and according to the factor loadings, most of the variables strongly influenced the factors, except for the variable *timework* (which corresponds to the instrument’s item “On average, how many hours per weekday do you spend working?”), which had a factor loading of 0.197, indicating a weak influence on the factor work strain. The pattern of correlations was generally low (all correlations below 0.50, see Supplementary Table S2) and coherent with the theoretical framework. Still, there was a statistically significant correlation between independence and work strain, independence and emotional intelligence, work strain and discrimination, and social support and emotional intelligence (all positive correlations).

### 3.3 Logistic and linear regressions

Each regression model was performed twice, either with sex or with gender as a covariate (in addition to the covariates age and years of education). However, since the results were practically the same, only the models using sex as covariate are presented in the main manuscript, while the models using gender can be found at Supplementary Tables S3, S4. Moreover, a heatmap is shown in Supplementary Figure S1 to visually represent the pattern of associations between all continuous variables.

**Figure 2:**
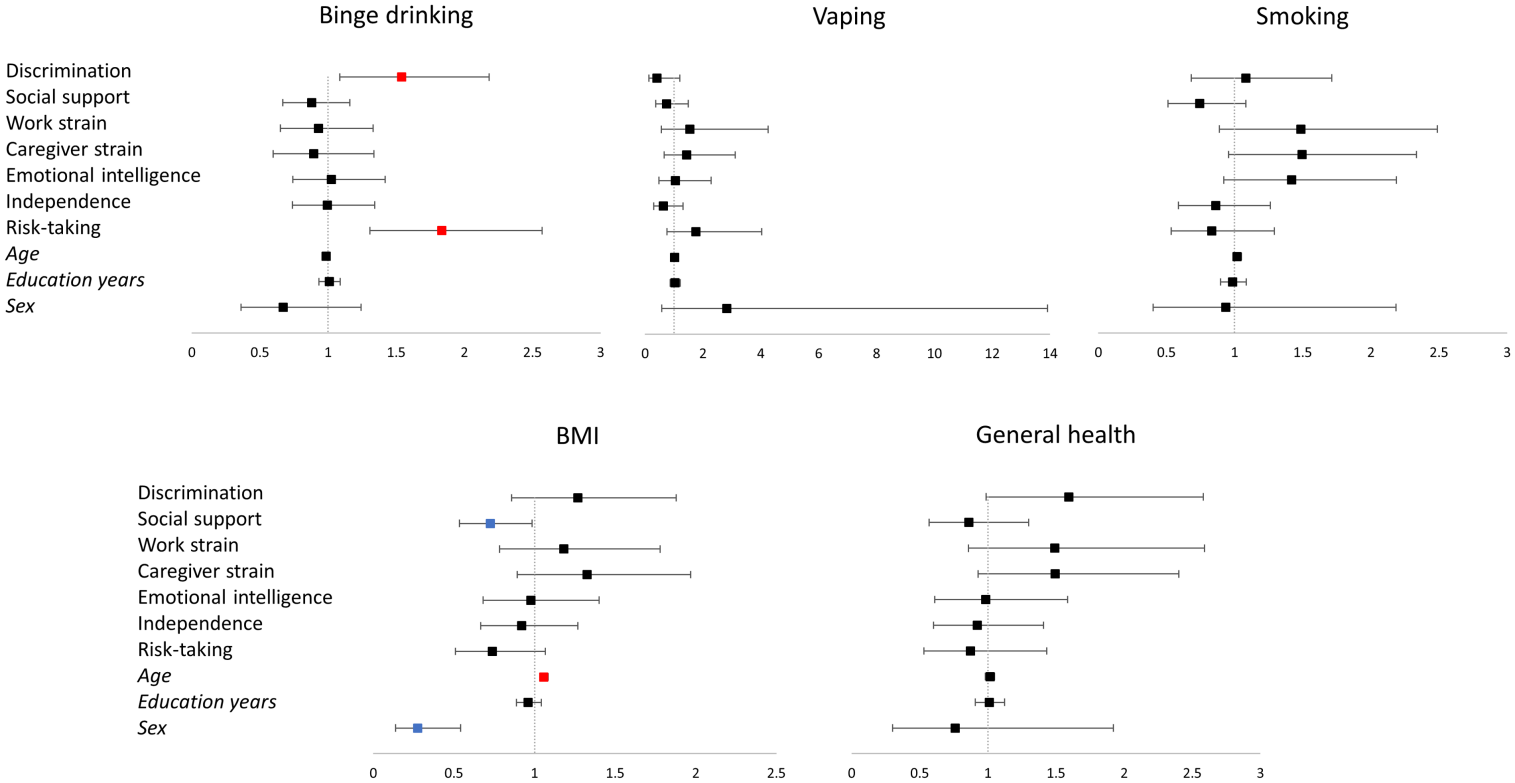
Representation of the logistic regression models for binge drinking, vaping, smoking, BMI, and general health. The vertical line corresponds to the boundary of statistical significance (i.e., no effect). Each row represents each of the predictors shown on the left. The squares represent the odds ratio, and the 95% confidence interval is represented as the segment line; significant effects are colored in red for positive and in blue for negative effects. Categorical variables codification: Binge drinking (less than monthly = 0; monthly, weekly, or daily = 1); Vaping (no = 0; yes = 1); Smoking (no = 0; =1); BMI (BMI < 25 = 0; BMI ≥ 25 = 1); General health (good, very good, excellent = 0; fair, poor = 1); Sex (male = 0; female = 1). BMI, Body mass index.

Logistic regression models of vaping (χ^2^ = 9.898; *p* = 0.449; McFadden’s *R*^2^ = 0.095), smoking (χ^2^ = 15.091; *p* = 0.129; McFadden’s *R*^2^ = 0.054), and general health (χ^2^ = 12.624; *p* = 0.245; McFadden’s *R*^2^ = 0.052) were not statistically significant, while associations were significant for binge drinking (χ^2^ = 28.436; *p* = 0.002; McFadden’s *R*^2^ = 0.063) and BMI (χ^2^ = 52.85; *p* < 0.001; McFadden’s *R*^2^ = 0.13). More specifically, higher levels of discrimination and risk-taking were associated with binge drinking, while lower social support, older age, and male sex were associated with more likelihood of being overweight or obese (see Table 2; Figure 2).

**Table 2:** Odds ratios of associations with binge drinking, vaping, smoking, BMI and general health measures in logistic regressions.

	Binge drinking	Vaping	Smoking	BMI	General health
Discrimination	1.540* (1.087; 2.183)	0.408 (0.138; 1.208)	1.082 (0.682; 1.714)	1.269 (0.857; 1.878)	1.595 (0.986; 2.582)
Social support	0.880 (0.667; 1.161)	0.742 (0.370; 1.489)	0.744 (0.510; 1.084)	0.725* (0.534; 0.986)	0.860 (0.569; 1.299)
Work strain	0.930 (0.650; 1.331)	1.543 (0.559; 4.255)	1.485 (0.887; 2.488)	1.181 (0.784; 1.779)	1.491 (0.858; 2.590)
Caregiver strain	0.894 (0.598; 1.336)	1.433 (0.658; 3.118)	1.494 (0.957; 2.335)	1.326 (0.894; 1.968)	1.493 (0.929; 2.400)
Emotional intelligence	1.024 (0.740; 1.418)	1.046 (0.479; 2.284)	1.419 (0.921; 2.187)	0.977 (0.681; 1.401)	0.984 (0.611; 1.585)
Independence	0.994 (0.737; 1.342)	0.627 (0.301; 1.308)	0.862 (0.588; 1.262)	0.920 (0.667; 1.269)	0.921 (0.602; 1.408)
Risk-taking	1.834* (1.308; 2.571)	1.755 (0.764; 4.030)	0.832 (0.535; 1.293)	0.738 (0.510; 1.068)	0.872 (0.531; 1.432)
Age	0.985 (0.961; 1.010)	1.016 (0.962; 1.074)	1.019 (0.989; 1.050)	1.058* (1.032; 1.085)	1.016 (0.982; 1.050)
Education years	1.009 (0.933; 1.090)	1.029 (0.870; 1.219)	0.986 (0.896; 1.085)	0.961 (0.887; 1.041)	1.011 (0.908; 1.124)
Sex	0.670 (0.362; 1.241)	2.826 (0.574; 13.910)	0.936 (0.401; 2.184)	0.275* (0.139; 0.542)	0.760 (0.301; 1.920)
Constant	0.853 (0.223; 3.267)	0.004 (0; 0.112)	0.104* (0.017; 0.628)	0.272 (0.064; 1.151)	0.07* (0.009; 0.513)

The values in parentheses correspond to the 95% confidence intervals. *Statistically significant association (*p* < 0.05).

Categorical variables codification: Binge drinking (less than monthly = 0; monthly, weekly, or daily = 1); Vaping (no = 0; yes = 1); Smoking (no = 0; yes = 1); BMI (BMI < 25 = 0; BMI ≥ 25 = 1); General health (good, very good, excellent = 0; fair, poor = 1); Sex (male = 0; female = 1).

**Figure 3:**
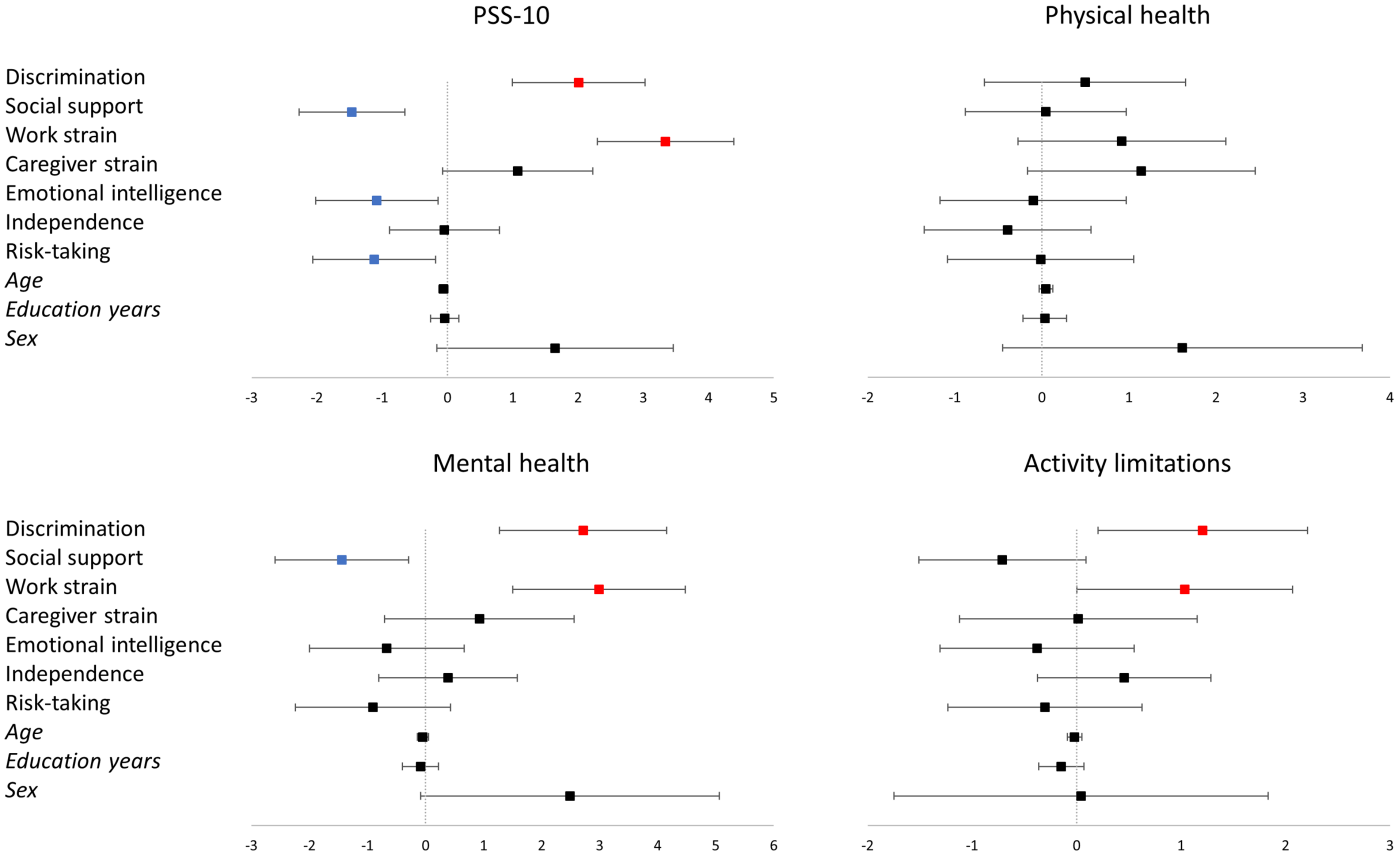
Representation of the linear regression models for PSS-10, physical health, mental health, and activity limitations. The vertical line corresponds to the boundary of statistical significance (i.e., no effect). Each row represents each of the predictors shown on the left. The squares represent the regression coefficients, and the 95% confidence interval is represented as the segment line; significant effects are colored in red for positive and in blue for negative effects. Categorical variables codification: Sex (male = 0; female = 1). PSS-10: Perceived Stress Scale.

Linear regression models of PSS-10 (*F* = 15.549; *p* < 0.001; *R*^2^ = 0.314), mental health (*F* = 9.015; *p* < 0.001; *R*^2^ = 0.210) and activity limitations (*F* = 3.142; *p* < 0.001; *R*^2^ = 0.085) were statistically significant, while the model of physical health (*F* = 1.671; *p* = 0.086; *R*^2^ = 0.047) was not. Regarding PSS-10, discrimination and work strain were significant positive predictors, while higher social support, emotional intelligence and risk-taking were significant negative predictors. Concerning mental health, discrimination and work strain were significant positive predictors (of poorer mental health), while social support was a significant negative predictor. Finally, for activity limitations, discrimination and work strain were significant positive predictors (Table 3; Figure 3).

**Table 3:** Unstandardized beta coefficients of associations with PSS-10, physical health, mental health and activity limitations measures in linear regressions.

	PSS-10	Physical health	Mental health	Activity limitations
Discrimination	2.013* (0.508)	0.496 (0.579)	2.716* (0.721)	1.208* (0.502)
Social support	−1.464* (0.405)	0.046 (0.462)	−1.444* (0.575)	−0.711 (0.400)
Work strain	3.340* (0.523)	0.920 (0.596)	2.992* (0.743)	1.036* (0.517)
Caregiver strain	1.077 (0.575)	1.144 (0.655)	0.928 (0.817)	0.017 (0.568)
Emotional intelligence	−1.082* (0.469)	−0.099 (0.535)	−0.668 (0.667)	−0.378 (0.464)
Independence	−0.046 (0.420)	−0.392 (0.479)	0.385 (0.597)	0.457 (0.415)
Risk-taking	−1.124* (0.470)	−0.014 (0.536)	−0.907 (0.668)	−0.303 (0.465)
Age	−0.062 (0.034)	0.047 (0.039)	−0.047 (0.049)	−0.019 (0.034)
Education years	−0.041 (0.109)	0.035 (0.125)	−0.088 (0.155)	−0.147 (0.108)
Sex	1.648 (0.906)	1.614 (1.033)	2.488 (1.287)	0.042 (0.896)
Constant	30.538* (1.957)	1.31 (2.232)	11.777* (2.781)	6.761* (1.934)

The values in parentheses correspond to the standard error. *Statistically significant association (*p* < 0.05).

PSS-10: Perceived Stress Scale. Physical/Mental health refer to the number of days with poor physical/mental health during the last 30 days, while Activity limitations refers to the number of days with activity limitations due to poor physical or mental health during the last 30 days.

Categorical variables codification: Sex (male = 0; female = 1).

### 3.4 Regression models in two subsamples of the data

Due to the sex unbalance found in our sample, and the lack of representativity of our age distribution when compared with the general Portuguese population, we decided to repeat the multiple regression models in two subsamples of the data: subsample 1 included 100 females and 100 males matched on age (mean 32.41, SD 12.64) and education level (mean 17.17, SD 3.52); and subsample 2 consisted on a sample more representative of the population also regarding age, including 50 females and 50 males matched on age (mean 39.77, SD 13.45) and education level (mean 17.35, SD 4.03). These results should be taken with caution due to the underpowered sample size, and are only intended to complement the full sample results, and provide some insights into the potential effects of the lack of representativeness of our data.

In subsample 1, the findings were almost the same as with the full sample. Logistic regression models of binge drinking, vaping, smoking, and general health were not statistically significant, while the BMI model was statistically significant. As happened with the full sample, lower social support, older age, and male sex were associated with more likelihood of being overweight or obese (Supplementary Table S5). Regarding linear regression models, those of PSS-10, mental health and activity limitations were statistically significant, while the model of physical health was not. For PSS-10, discrimination and work strain were significant positive predictors, while higher social support was a significant negative predictor. Concerning mental health, work strain was a significant positive predictor (of poorer mental health), while social support was a significant negative predictor. Finally, for activity limitations, social support was a significant negative predictor (Supplementary Table S6).

The results from subsample 2, on the other hand, differed greater from those from the full sample, which could be partly due to the loss of statistical power, in addition to the different characteristics of the sample. None of the logistic regression models were statistically significant (the Vaping model could not even be performed due to only 3 participants categorized as vaping users) (Supplementary Table S7), while the linear regression models of PSS-10, physical health and mental health were statistically significant (for activity limitations the *p*-value of the model was 0.064). More specifically, social support was a significant negative predictor of PSS-10 and mental health, while caregiver strain was a significant positive predictor of physical health (Supplementary Table S8).

## 4 Discussion

In general, and according to the values obtained in the CFA, we can consider that the factor structure of the original GVHR scale was successfully replicated in the Portuguese population (Boateng et al., 2018). Most specific factor loadings were appropriate for all factors, with the exception of *timework*. However, we believe this reflects the characteristics of our sample rather than a general issue with the scale’s factor structure. Specifically, regarding the sample distribution on professional status, there was a high percentage of participants not working (57.8%), which is not representative of the general population (unlike the sample used in the original paper). The sample included in the Spanish validation of the GVHR by Díaz-Morales et al. (2023) had a higher percentage of people working (56.8%), but they did not include the *timework* item in their work strain factor, so we cannot use their sample for comparison. In any case, future studies may attempt to replicate our findings on a more representative sample to check if a more appropriate factor loading for *timework* is achieved.

Regarding the association between gender-related variables and health outcomes, as in the original publication, higher risk-taking was associated with binge drinking. The study by de Haan et al. also showed that risk-taking was significantly related to alcohol use in both men and women after controlling for age, lifestyle, depression, anxiety, and stress levels, supporting our results (de Haan et al., 2015). One potential explanation for this association is that individuals with a higher propensity for risk-taking tend to engage in behaviors that provide immediate gratification, including alcohol consumption, without fully considering long-term health consequences (Henges and Marczinski, 2012). Furthermore, impulsivity, which is closely related to risk-taking, has been identified as a key factor in substance use behaviors (Stautz and Cooper, 2013).

Additionally, we found that gender discrimination was associated with binge drinking. This aligns with findings from a systematic review by Gilbert and Zemore (2016) on discrimination based on race, sexual orientation, and gender, which suggested that discrimination contributes to stress-related coping mechanisms such as increased alcohol use. This relationship can be explained through the stress-coping model, which posits that individuals experiencing discrimination may use substances as a way to manage psychological distress (Hatzenbuehler, 2009). In this line, previous studies have documented that mental health and well-being can also be influenced by various forms of discrimination, including gender discrimination, which is related to stress, anxiety, and depressive symptoms (Perry et al., 2013). According to our outcomes, gender discrimination arises as a significant predictor of mental health worsening and activity limitations, in line with the results of Nielsen et al. (2021) and Díaz-Morales et al. (2023). Additionally, it was also significantly associated with higher perceived stress. Interestingly, when repeating these analyses in subsample 2, which included fewer younger participants and had an age distribution more similar to that of the general Portuguese population, the discrimination factor was no longer significantly associated with any health outcome. This could be indicating that young people are more aware about their gender discrimination experiences, making this factor more relevant for their health outcomes compared to older participants.

Regarding BMI, as in the original paper and the Spanish validation, being male was associated with an increased likelihood of being overweight, while we additionally found an association with lower social support and older age. Social support has been well established as a protective factor for health outcomes, including weight management, likely due to its role in facilitating healthy behaviors, reducing stress, and promoting self-efficacy (Arigo et al., 2021; Karfopoulou et al., 2016; Kim et al., 2021). Similarly, age-related weight gain has been documented in various studies, in line with our findings, as metabolic rate decreases and lifestyle factors such as decreased physical activity contribute to increased obesity risk (Gao et al., 2023; Palmer and Jensen, 2022; Shakya et al., 2023).

Another factor that was shown to be relevant for health outcomes was work strain, which was associated with higher perceived stress, mental health worsening and activity limitations. This result is consistent with the findings from the Spanish validation of the GVHR scale (Díaz-Morales et al., 2023), and with previous literature showing that jobs associated with higher strain and tension were related to more psychological distress in both men and women, due to a higher psychological demand (Vermeulen and Mustard, 2000). Notably, the work strain factor included questions about how emotionally and physically exhausted participants felt from their work activities, which referred to both job-related and student-related activities. This could be particularly relevant in our sample, which we suspect may include many medical students because of our sampling procedure (although we distributed the study across all the university and the general community, our research group is situated in the School of Medicine, which probably had an influence on students’ participation). Several studies have shown that medical students experience high stress levels during their training (Kötter et al., 2017; Ragab et al., 2021), with almost half of the students attending the sixth year of Portuguese medical schools have pathological stress (Oura et al., 2020). Regrettably, although we do know that 55% of our participants were students, we did not collect information on the degree of study, so future studies focused on this population could better characterize the specific risks associated with the degree of study.

On the other hand, we found that social support had a positive influence on well-being, being a negative predictor of perceived stress and mental health worsening. Our results seem to be aligned with those found in the Spanish validation of the scale, as well as with previous studies showing that social support and stress impact health in opposite directions, and that social isolation (the opposite of social support) is a risk factor for mortality (Holt-Lunstad et al., 2015; Kneavel, 2020; Lee and Dik, 2017). Lastly, emotional intelligence also appeared as a protective factor against stress. Emotional intelligence corresponds to the ability to perceive, evaluate, and manage one’s own emotions and the emotions of others. The relationship between this variable and the ability to deal with stressful moments has been well described in the literature (Fteiha and Awwad, 2020; Sharma and Kumar, 2016), and our results go in accordance with previous findings.

Regarding the dissimilarities between this study and the original paper as well as the Spanish validation, we found no associations with the caregiver strain factor, which was a significant predictor of worse health outcomes in those populations. Our lack of associations might be explained by the large number of single participants in our sample (71.2%), making the weight of the caregiver strain variable less relevant. Furthermore, unlike in the original paper, none of our gender-related variables had an influence on physical health. The fact that we had a generally young sample, with an average age of 30.4, may not yet reflect the negative impact on physical health that these variables could eventually have, being at this point in life only relevant for mental health and activity limitations. This seems to be supported by our complementary analyses performed in subsample 2, which had fewer younger participants and included an age distribution more similar to that of the general Portuguese population (mean age = 39.77). For that subsample, higher caregiver strain was significantly associated with worse physical health, in line with the results from Nielsen et al. (2021) and Díaz-Morales et al. (2023). Another difference, specifically with the Spanish validation, had to do with the emotional intelligence factor. While it did not show good reliability in the Spanish validation, and thus was removed from their version of the scale, it performed well in our dataset, and appeared to be a protective factor against stress.

Finally, our findings have important practical implications, particularly for mental health prevention and intervention strategies. Given the significant associations between gender discrimination, social support, and stress, anti-discrimination policies are crucial, including workplace and educational equity programs. Social support initiatives to mitigate stress-related health risks, especially for populations at risk of social isolation, would also be beneficial. In clinical settings, screening for gender-related vulnerability factors and providing tailored psychological support may be helpful in adjusting psychotherapeutic approaches and preventing further mental health deterioration. For example, since emotional intelligence emerged as a protective factor, integrating emotional intelligence training into stress management programs could enhance coping strategies.

Some limitations of this study should be considered. Firstly, our complete sample was not representative of the general Portuguese population (Instituto Nacional de Estatística, 2022), with a mean age of 30.4 (versus 46.7), a 71.5% of females (versus 52.3%), or a highly educated sample, for example. Thus, this could have prevented some of our factor loadings to achieve better values (*timework*, *indepgen*, and *discrhire*). Future studies should seek to replicate our results in larger, more diverse populations to improve generalizability. Nonetheless, we repeated the regression analyses using subsamples of the data more representative of the general population, and the main findings seem to remain significant, although these results should be taken with caution given their post-hoc nature and the loss of statistical power. Additionally, given our cross-sectional design, longitudinal research is needed to establish causal relationships between gender-related variables and health outcomes. Exploring these associations over time could provide stronger evidence for targeted interventions. Finally, we measured health-related variables (general health, mental health, physical health, and activities limitations) using a specific retrospective (last 30 days) instrument, following the same approach as in the original study, but future studies could also try to associate gender-related variables with health outcomes measured in a more ecological way.

The validation of this scale represents the starting point for a multidimensional approach to gender in health research and clinical practice in the Portuguese population. Importantly, from the different health-related measures, gender-related variables were mainly associated with mental health-related variables (PSS-10, mental health, and activities limitations), highlighting the importance of considering gender variables when approaching mental health, including stress, in health research and clinical practice. Moreover, gender discrimination and social support seem to be two of the most relevant variables for health outcomes, highlighting the importance of the relational dimension of gender, which is typically ignored when approaching gender from an individual perspective alone. Finally, our results do not allow us to conclude any causality between the variables, so it would be interesting for future studies to longitudinally explore the association between gender-related variables and mental health variables, particularly stress perception. Expanding our knowledge about this relationship could have an impact in the preventive and therapeutic approach to stress in the clinical practice.

## Data Availability

The raw data supporting the conclusions of this article will be made available by the authors, without undue reservation.
