# Correction: Exploring the impact of gender-related variables on health measures and perceived stress

**DOI:** 10.3389/fpsyg.2026.1824746

**Published:** 2026-04-09

**Authors:** Maria Picó-Pérez, Marisa S. Coelho, Rita Vieira, Mafalda Machado-Sousa, Pedro Morgado

**Affiliations:** 1 Life and Health Sciences Research Institute (ICVS), University of Minho, Braga, Portugal; 2 ICVS/3B's, PT Government Associate Laboratory, Braga, Portugal; 3 Departamento de Psicología Básica, Clínica y Psicobiología, Universitat Jaume I, Castellón de la Plana, Spain; 4 Clinical Academic Center - Braga, Braga, Portugal

**Keywords:** gender, stress, mental health, discrimination, social support

There was a mistake in Figures 1, 2, and 3, and Tables 2 and 3 as published. The items of the factor independence of the GVHR scale were mistakenly reversed. This did not influence the confirmatory factor analysis or any of the main findings (considering there were no significant results for the regression models), but the sign of the beta coefficients and the values of the odds ratios were reversed once this was corrected, influencing all tables and figures where these values were shown. This correction did not affect figure/table captions or their order. The corrected Figures and Tables appear below.

**Figure 1:**
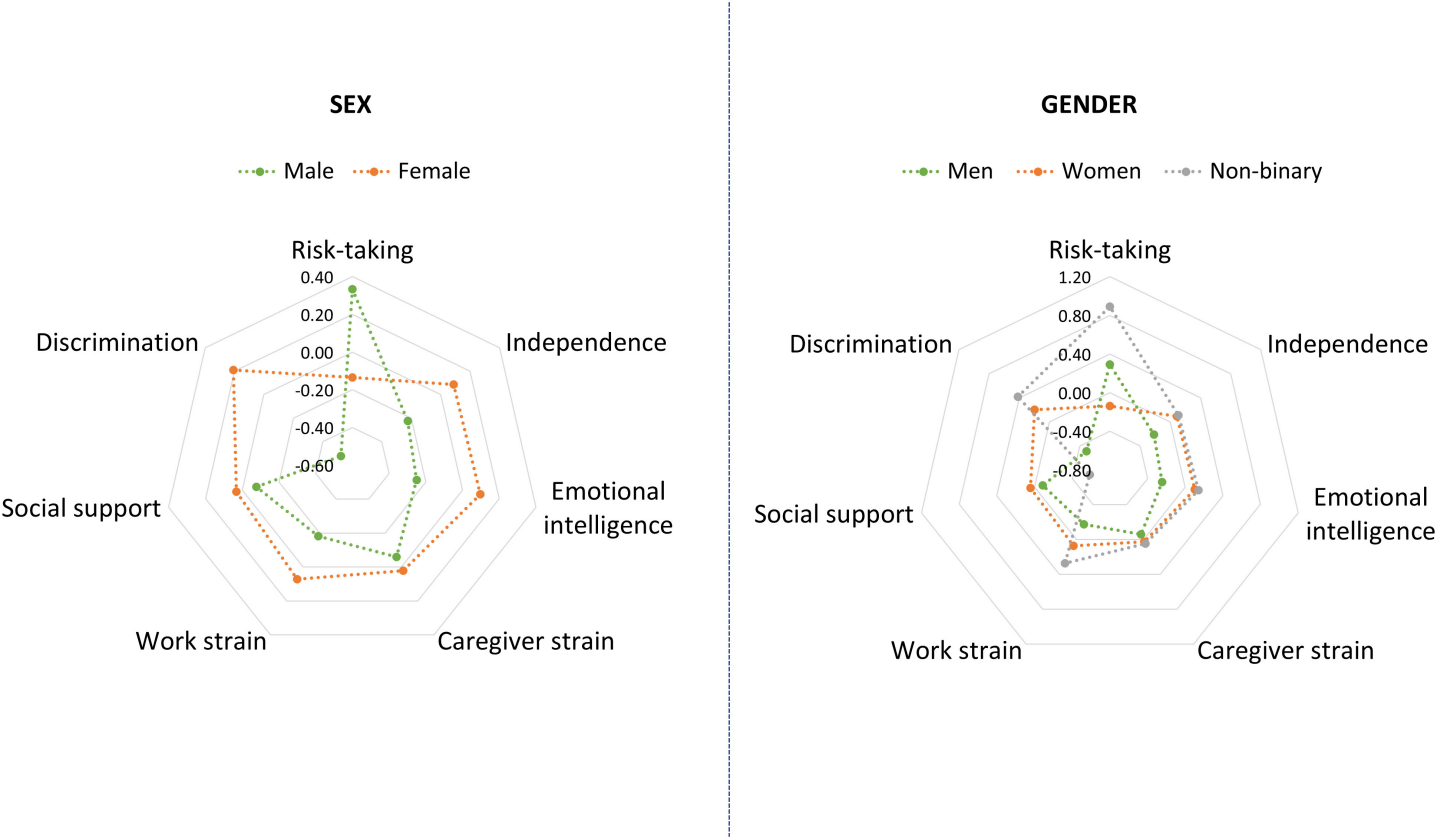
Mean *z*-scores for the seven GVHR factors, separately by sex **(left)** and by gender **(right)**.

**Figure 2:**
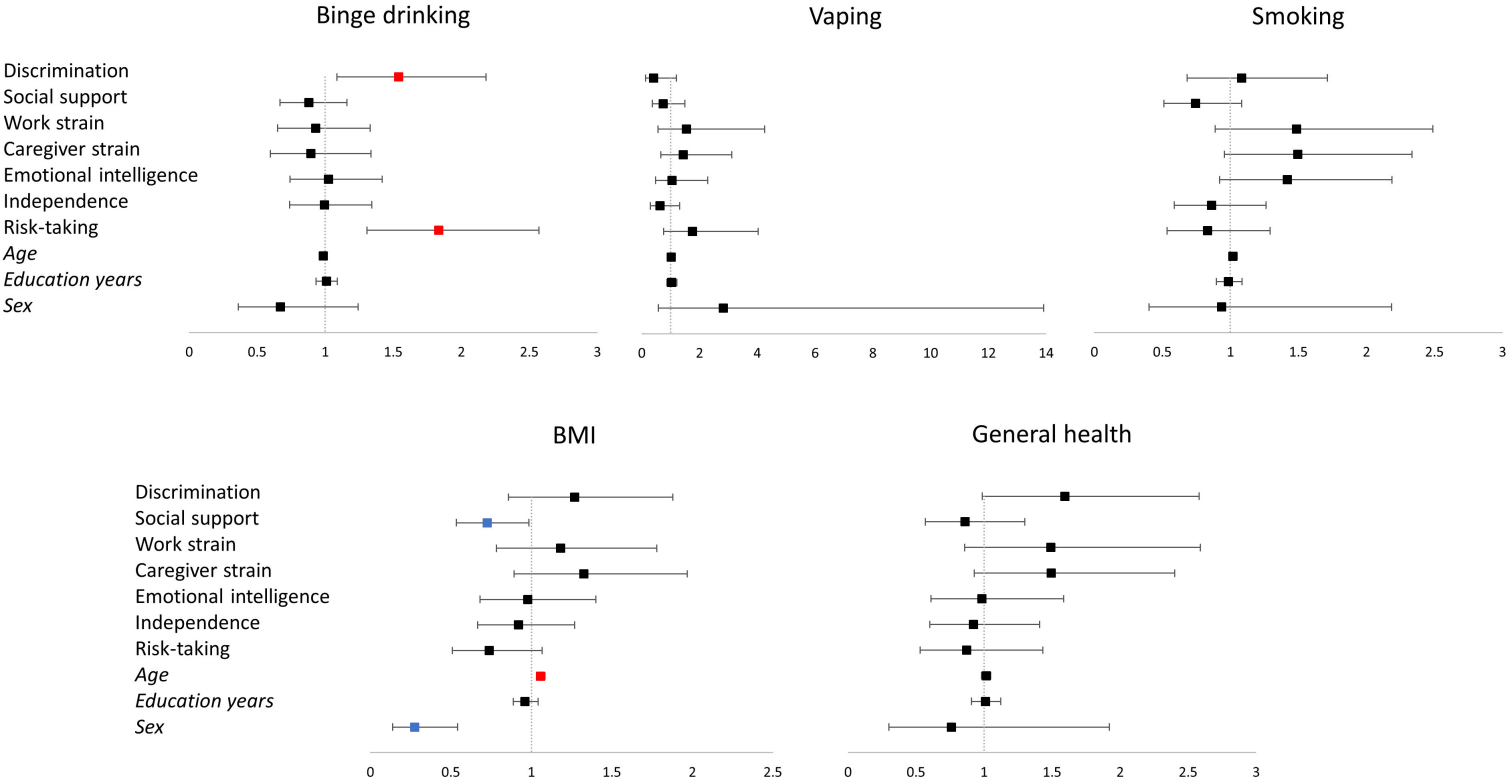
Representation of the logistic regression models for binge drinking, vaping, smoking, BMI, and general health. The vertical line corresponds to the boundary of statistical significance (i.e., no effect). Each row represents each of the predictors shown on the left. The squares represent the odds ratio, and the 95% confidence interval is represented as the segment line; significant effects are colored in red for positive and in blue for negative effects. Categorical variables codification: Binge drinking (less than monthly = 0; monthly, weekly, or daily = 1); Vaping (no = 0; yes = 1); Smoking (no = 0; =1); BMI (BMI < 25 = 0; BMI ≥ 25 = 1); General health (good, very good, excellent = 0; fair, poor = 1); Sex (male = 0; female = 1). BMI, body mass index.

**Figure 3:**
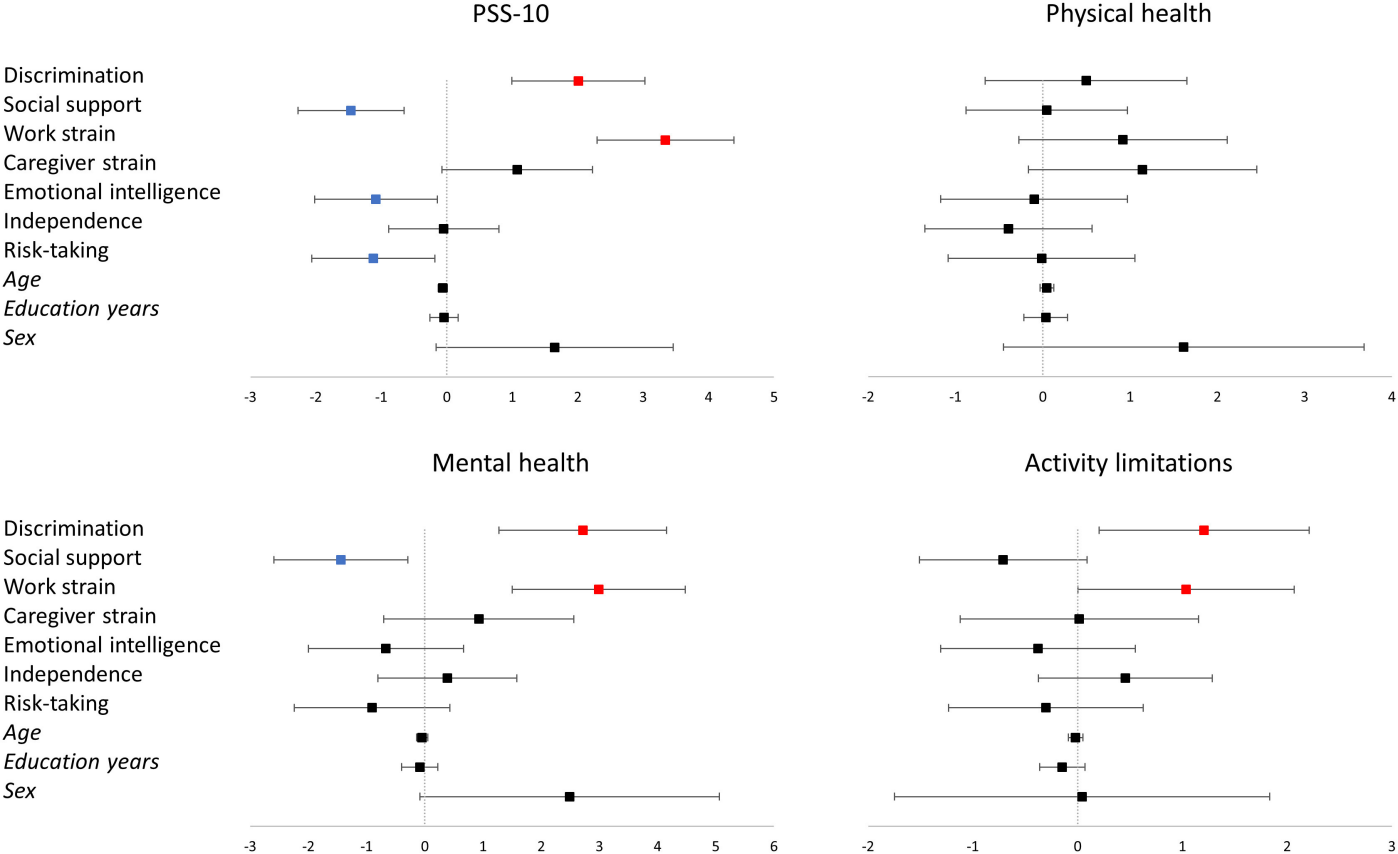
Representation of the linear regression models for PSS-10, physical health, mental health, and activity limitations. The vertical line corresponds to the boundary of statistical significance (i.e., no effect). Each row represents each of the predictors shown on the left. The squares represent the regression coefficients, and the 95% confidence interval is represented as the segment line; significant effects are colored in red for positive and in blue for negative effects. Categorical variables codification: Sex (male = 0; female = 1). PSS-10: Perceived Stress Scale.

**Table 2:** Odds ratios of associations with binge drinking, vaping, smoking, BMI and general health measures in logistic regressions.

	Binge drinking	Vaping	Smoking	BMI	General health
Discrimination	1.540^*^ (1.087; 2.183)	0.408 (0.138; 1.208)	1.082 (0.682; 1.714)	1.269 (0.857; 1.878)	1.595 (0.986; 2.582)
Social support	0.880 (0.667; 1.161)	0.742 (0.370; 1.489)	0.744 (0.510; 1.084)	0.725^*^ (0.534; 0.986)	0.860 (0.569; 1.299)
Work strain	0.930 (0.650; 1.331)	1.543 (0.559; 4.255)	1.485 (0.887; 2.488)	1.181 (0.784; 1.779)	1.491 (0.858; 2.590)
Caregiver strain	0.894 (0.598; 1.336)	1.433 (0.658; 3.118)	1.494 (0.957; 2.335)	1.326 (0.894; 1.968)	1.493 (0.929; 2.400)
Emotional intelligence	1.024 (0.740; 1.418)	1.046 (0.479; 2.284)	1.419 (0.921; 2.187)	0.977 (0.681; 1.401)	0.984 (0.611; 1.585)
Independence	0.994 (0.737; 1.342)	0.627 (0.301; 1.308)	0.862 (0.588; 1.262)	0.920 (0.667; 1.269)	0.921 (0.602; 1.408)
Risk-taking	1.834^*^ (1.308; 2.571)	1.755 (0.764; 4.030)	0.832 (0.535; 1.293)	0.738 (0.510; 1.068)	0.872 (0.531; 1.432)
Age	0.985 (0.961; 1.010)	1.016 (0.962; 1.074)	1.019 (0.989; 1.050)	1.058^*^ (1.032; 1.085)	1.016 (0.982; 1.050)
Education years	1.009 (0.933; 1.090)	1.029 (0.870; 1.219)	0.986 (0.896; 1.085)	0.961 (0.887; 1.041)	1.011 (0.908; 1.124)
Sex	0.670 (0.362; 1.241)	2.826 (0.574; 13.910)	0.936 (0.401; 2.184)	0.275^*^ (0.139; 0.542)	0.760 (0.301; 1.920)
Constant	0.853 (0.223; 3.267)	0.004 (0; 0.112)	0.104^*^ (0.017; 0.628)	0.272 (0.064; 1.151)	0.07^*^ (0.009; 0.513)

The values in parentheses correspond to the 95% confidence intervals.

^*^Statistically significant association (*p* < 0.05).

Categorical variables codification: Binge drinking (less than monthly = 0; monthly, weekly, or daily = 1); Vaping (no = 0; yes = 1); Smoking (no = 0; yes = 1); BMI (BMI < 25 = 0; BMI ≥ 25 = 1); General health (good, very good, excellent = 0; fair, poor = 1); Sex (male = 0; female = 1).

**Table 3:** Unstandardized beta coefficients of associations with PSS-10, physical health, mental health and activity limitations measures in linear regressions.

	PSS-10	Physical health	Mental health	Activity limitations
Discrimination	2.013^*^ (0.508)	0.496 (0.579)	2.716^*^ (0.721)	1.208^*^ (0.502)
Social support	−1.464^*^ (0.405)	0.046 (0.462)	−1.444^*^ (0.575)	−0.711 (0.400)
Work strain	3.340^*^ (0.523)	0.920 (0.596)	2.992^*^ (0.743)	1.036^*^ (0.517)
Caregiver strain	1.077 (0.575)	1.144 (0.655)	0.928 (0.817)	0.017 (0.568)
Emotional intelligence	−1.082^*^ (0.469)	−0.099 (0.535)	−0.668 (0.667)	−0.378 (0.464)
Independence	−0.046 (0.420)	−0.392 (0.479)	0.385 (0.597)	0.457 (0.415)
Risk-taking	−1.124^*^ (0.470)	−0.014 (0.536)	−0.907 (0.668)	−0.303 (0.465)
Age	−0.062 (0.034)	0.047 (0.039)	−0.047 (0.049)	−0.019 (0.034)
Education years	−0.041 (0.109)	0.035 (0.125)	−0.088 (0.155)	−0.147 (0.108)
Sex	1.648 (0.906)	1.614 (1.033)	2.488 (1.287)	0.042 (0.896)
Constant	30.538^*^ (1.957)	1.31 (2.232)	11.777^*^ (2.781)	6.761^*^ (1.934)

The values in parentheses correspond to the standard error.

^*^Statistically significant association (*p* < 0.05).

PSS-10: Perceived Stress Scale. Physical/Mental health refer to the number of days with poor physical/mental health during the last 30 days, while Activity limitations refers to the number of days with activity limitations due to poor physical or mental health during the last 30 days.

Categorical variables codification: Sex (male = 0; female = 1).

File “Supplementary Table 1” was erroneously published with the original version of this paper. This file contains errors, due to the same mistake with the reverse coding of the independence factor of the GVHR scale described for the figures and tables. This affects Supplementary Figure 1 and Supplementary Tables 2–8. The file has now been replaced.

The items of the factor independence of the GVHR scale were mistakenly reversed. This did not influence the confirmatory factor analysis or any of the main findings (considering there were no significant results for the regression models), but the sign of the beta coefficients and the values of the odds ratios were reversed once this was corrected (which affected the figures, tables, and supplementary material). Furthermore, if influenced the direction of the correlations between factors described in the Results.

A correction has been made to **Section 3. Results**, *3.2. Confirmatory factor analysis*:

“CFA of GVHR indicated acceptable fit, with χ^2^ = 456.058, χ^2^/df ratio = 1.788, CFI = 0.942, GFI = 0.903, TLI = 0.931, and RMSEA = 0.047. Also, all factors showed appropriate internal consistency (see Supplementary Table 1 for their Cronbach's alpha coefficients), and according to the factor loadings, most of the variables strongly influenced the factors, except for the variable timework (which corresponds to the instrument's item “On average, how many hours per weekday do you spend working?”), which had a factor loading of 0.197, indicating a weak influence on the factor work strain. The pattern of correlations was generally low (all correlations below 0.50, see Supplementary Table 2) and coherent with the theoretical framework. Still, there was a statistically significant correlation between independence and work strain, independence and emotional intelligence, work strain and discrimination, and social support and emotional intelligence (all positive correlations).”

A correction has been made to section **2. Materials and methods**, *2.4. Statistical analysis, third paragraph*:

“Regarding the GVHR questionnaire, we recoded the missing data into 1 for the variables caregiver strain and work strain, following the same approach as in the original study. That is, people not currently caring for someone in need or not currently employed (and thus not responding to the caregiver strain and work strain questions) were ascribed the value 1, which represents no strain due to caregiving/work. Finally, we calculated standardized z-scores for each variable from the GVHR questionnaire, and mean-item subscale scores were computed for each of the seven factors, to be used later as predictors in the regression analyses. All variables are scored from lower to higher levels of the given constructs. Figure 1 displays the mean z-scores for the seven GVHR factors, separately by sex as well as by gender.”

The original version of this article has been updated.

