# Supplementary material for: Exploring the impact of gender-related variables on health measures and perceived stress
Source: Front Psychol. 2025 Feb 25;16:1500674. doi: 10.3389/fpsyg.2025.1500674 (PMC11893839; doi:10.3389/fpsyg.2025.1500674)
Supplement: Supplementary file 1 [file Table_1.docx]

Supplementary Material

**Supplementary Table 1.** Cronbach’s alpha and factor loadings for each factor of the CFA model.

| Factor | Cronbach’s alpha | Item | Factor loadings |
| --- | --- | --- | --- |
| Caregiver strain | .626 | careemot | 0.979 |
|  |  | carephys | 0.934 |
|  |  | carefut | 0.927 |
|  |  | timecare | 0.587 |
| Work strain | .707 | workspeed | 0.630 |
|  |  | workrep | 0.558 |
|  |  | workemot | 0.764 |
|  |  | workphys | 0.725 |
|  |  | timework | 0.197 |
| Independence | .600 | indepprob | 0.954 |
|  |  | indepgen | 0.450 |
| Risk-taking | .703 | riskgen | 0.910 |
|  |  | riskfinan | 0.510 |
|  |  | riskrecrea | 0.611 |
| Emotional intelligence | .643 | commfriend | 0.433 |
|  |  | exprfriend | 0.827 |
|  |  | exprother | 0.652 |
| Social support | .654 | socsuplove | 0.739 |
|  |  | socsupchores | 0.658 |
| Discrimination | .860 | discrgen | 0.842 |
|  |  | discrhire | 0.536 |
|  |  | discred | 0.775 |
|  |  | discrmed | 0.643 |
|  |  | discrpub | 0.857 |
|  |  | discrfam | 0.593 |

**Supplementary Table 2.** Pearson Correlation Coefficients between the factors of the Gender-Related Variables for Health Research.

|  | 1 | 2 | 3 | 4 | 5 | 6 | 7 |
| --- | --- | --- | --- | --- | --- | --- | --- |
| Caregiver strain (1) | 1.000 |  |  |  |  |  |  |
| Work strain (2) | 0.071 | 1.000 |  |  |  |  |  |
| Independence (3) | 0.104 | 0.199** | 1.000 |  |  |  |  |
| Risk-taking (4) | 0.034 | -0.157 | 0.204 | 1.000 |  |  |  |
| Emotional intelligence (5) | 0.088 | 0.087 | 0.211** | 0.156 | 1.000 |  |  |
| Social support (6) | 0.034 | -0.077 | 0.107 | 0.062 | 0.212** | 1.000 |  |
| Discrimination (7) | -0.076 | 0.293** | 0.094 | -0.029 | 0.117 | -0.141 | 1.000 |

**Correlations significant at the .01 level.

**Supplementary Figure 1.** Heatmap showing the pattern of associations between all continuous variables (the seven GVHR factors and the four health outcomes).


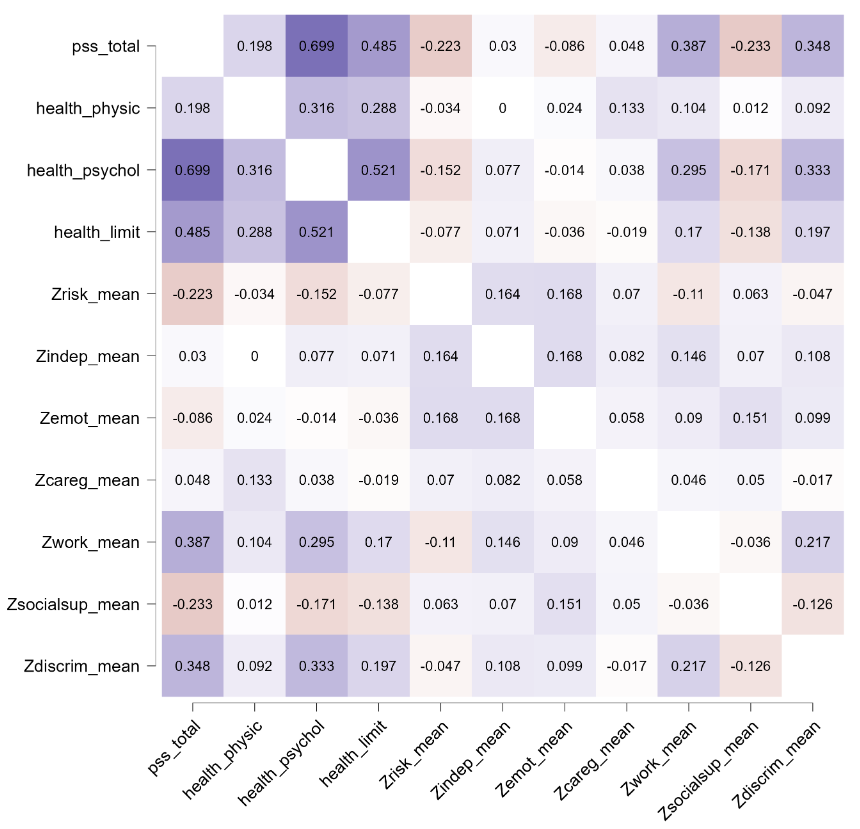


**Supplementary Table 3.** Odds ratios of associations with binge drinking, vaping, smoking, BMI and general health measures in logistic regressions, using gender instead of sex as a covariate.

|  | Binge drinking | Vaping | Smoking | BMI | General health |
| --- | --- | --- | --- | --- | --- |
| Discrimination | **1.492*** (1.058;2.105) | 0.417 (0.143;1.222) | 1.137 (0.722;1.791) | 1.206 (0.822;1.77) | 1.62  (1.009;2.599) |
| Social support | 0.873 (0.661;1.155) | 0.736 (0.367;1.476) | 0.749 (0.515;1.091) | **0.729*** (0.536;0.989) | 0.861  (0.57;1.3) |
| Work strain | 0.925 (0.645;1.326) | 1.552 (0.564;4.268) | 1.483 (0.885;2.484) | 1.181 (0.785;1.777) | 1.503 (0.864;2.613) |
| Caregiver strain | 0.884 (0.591;1.323) | 1.434 (0.661;3.109) | 1.518 (0.972;2.369) | 1.316 (0.888;1.951) | 1.506 (0.938;2.417) |
| Emotional intelligence | 1.019 (0.736;1.411) | 1.041 (0.475;2.282) | 1.454 (0.943;2.243) | 0.955 (0.668;1.367) | 0.985 (0.612;1.587) |
| Independence | 0.995 (0.736;1.343) | 0.628 (0.302;1.306) | 0.869 (0.595;1.271) | 0.910 (0.661;1.253) | 0.920 (0.602;1.406) |
| Risk-taking | **1.835*** (1.308;2.572) | 1.776 (0.772;4.085) | 0.826 (0.532;1.282) | 0.766 (0.531;1.104) | 0.889 (0.543;1.457) |
| Age | 0.987 (0.963;1.012) | 1.015 (0.961;1.073) | 1.018 (0.988;1.049) | **1.058*** (1.032;1.085) | 1.014 (0.981;1.048) |
| Education years | 1.009 (0.933;1.090) | 1.027 (0.867;1.217) | 0.982 (0.892;1.081) | 0.964 (0.89;1.044) | 1.009 (0.906;1.122) |
| Gender (woman) | 0.737 (0.404;1.344) | 2.863 (0.585;14) | 0.741 (0.332;1.654) | **0.315*** (0.164;0.604) | 0.7  (0.29;1.691) |
| Gender (non-binary) | 2.238 (0.213;23.545) | 4.095e-6  (0;∞) | 8.866e-7  (0;∞) | 0.666 (0.063;7.078) | 3.729e-7  (0;∞) |
| Constant | 0.732 (0.192;2.795) | **0.005***  (0;0.122) | **0.138***  (0.023;0.812) | **0.231*** (0.055;0.966) | **0.080***  (0.011;0.571) |
| χ² (p); McFadden’s R^2^ | **28.651 (.003); 0.064** | 10.188 (.514); 0.097 | 16.611 (.12); 0.059 | **50.534 (<.001); 0.125** | 13.812 (.244); 0.056 |

The values in parentheses for the predictors correspond to the 95% confidence intervals. *Statistically significant association (p < .05).

Categorical variables codification: Binge drinking (less than monthly=0; monthly, weekly, or daily=1); Vaping (no=0; yes=1); Smoking (no=0; yes=1); BMI (BMI<25=0; BMI≥25=1); General health (good, very good, excellent=0; fair, poor=1); Gender (man=0; woman=1; non-binary=2; one person replying “prefer not to say” was excluded from the analysis).

**Supplementary Table 4.** Unstandardized beta coefficients of associations with PSS-10, physical health, mental health and activity limitations measures in linear regressions, using gender instead of sex as a covariate.

|  | PSS-10 | Physical health | Mental health | Activity limitations |
| --- | --- | --- | --- | --- |
| Discrimination | **1.975*** (0.502) | 0.599 (0.574) | **2.791*** (0.714) | **1.149*** (0.496) |
| Social support | **-1.512*** (0.406) | 0.055 (0.465) | **-1.501*** (0.578) | -0.759 (0.402) |
| Work strain | **3.341*** (0.523) | 0.929 (0.599) | **3.014*** (0.745) | **1.054*** (0.518) |
| Caregiver strain | 1.062 (0.574) | 1.174 (0.658) | 0.953 (0.818) | 0.013 (0.569) |
| Emotional intelligence | **-1.098*** (0.468) | -0.064 (0.536) | -0.64 (0.666) | -0.413 (0.463) |
| Independence | -0.052 (0.419) | -0.372 (0.48) | 0.401 (0.597) | 0.427 (0.415) |
| Risk-taking | **-1.085*** (0.469) | -0.05 (0.537) | -0.871 (0.668) | -0.177 (0.465) |
| Age | -0.06 (0.034) | 0.044 (0.039) | -0.049 (0.049) | -0.021 (0.034) |
| Education years | -0.044 (0.109) | 0.031 (0.125) | -0.101 (0.156) | -0.151 (0.108) |
| Gender (woman) | **1.931*** (0.882) | 1.271 (1.01) | 2.375 (1.256) | 0.4 (0.873) |
| Gender (non-binary) | 0.559 (3.267) | -1.108 (3.741) | -2.728 (4.65) | -3.305 (3.233) |
| Constant | **30.344*** (1.95) | 1.768 (2.233) | **12.159*** (2.776) | **6.659*** (1.93) |
| F (p); R^2^ | **14.32 (<.001); 0.296** | 1.45 (.149); 0.045 | **8.263 (<.001); 0.212** | **2.985 (<.001); 0.089** |

The values in parentheses for the predictors correspond to the standard error. *Statistically significant association (p < .05).

PSS-10: Perceived Stress Scale. Physical/Mental health refer to the number of days with poor physical/mental health during the last 30 days, while Activity limitations refers to the number of days with activity limitations due to poor physical or mental health during the last 30 days.

Categorical variables codification: Gender (man=0; woman=1; non-binary=2; one person replying “prefer not to say” was excluded from the analysis).

**Supplementary Table 5.** Odds ratios of associations with binge drinking, vaping, smoking, BMI and general health measures in logistic regressions, using a matched subsample of 100 females and 100 males.

|  | Binge drinking | Vaping | Smoking | BMI | General health |
| --- | --- | --- | --- | --- | --- |
| Discrimination | 1.484 (0.917;2.401) | 0.27 (0.048;1.514) | 0.903 (0.473;1.724) | 1.515 (0.891;2.576) | 1.376  (0.711;2.662) |
| Social support | 1.033 (0.726;1.469) | 1.115 (0.44;2.821) | 0.892 (0.542;1.47) | **0.64*** (0.436;0.94) | 0.78  (0.466;1.305) |
| Work strain | 1.116 (0.7;1.78) | 1.117 (0.3;4.157) | 1.477 (0.772;2.825) | 0.821 (0.491;1.374) | 1.483 (0.699;3.146) |
| Caregiver strain | 0.875 (0.521;1.469) | 0.704 (0.175;2.832) | 1.107 (0.591;2.074) | 1.498 (0.9;2.492) | 1.555 (0.852;2.838) |
| Emotional intelligence | 0.969 (0.625;1.504) | 0.678 (0.204;2.25) | 1.662 (0.926;2.983) | 0.956 (0.598;1.528) | 0.767 (0.403;1.46) |
| Independence | 0.980 (0.669;1.436) | 0.612 (0.274;1.369) | 0.693 (0.434;1.105) | 1.009 (0.677;1.504) | 0.808 (0.474;1.377) |
| Risk-taking | **1.587*** (1.037;2.429) | 1.375 (0.488;3.875) | 0.657 (0.354;1.219) | 0.68 (0.421;1.097) | 1.082 (0.578;2.025) |
| Age | 0.982 (0.952;1.012) | 1.012 (0.949;1.079) | 1.013 (0.976;1.052) | **1.048*** (1.017;1.08) | 1.014 (0.974;1.055) |
| Education years | 1.056 (0.952;1.172) | 0.958 (0.766;1.198) | 1.032 (0.905;1.176) | 1.019 (0.919;1.129) | 1.021 (0.885;1.178) |
| Sex | 0.604 (0.282;1.294) | 4.817 (0.615;37.727) | 1.064 (0.387;2.926) | **0.224*** (0.094;0.533) | 1.087  (0.362;3.264) |
| Constant | 0.463 (0.088;2.447) | 0.011  (0;1.003) | **0.054***  (0.005;0.557) | **0.139*** (0.024;0.821) | **0.048***  (0.004;0.61) |
| χ² (p); McFadden’s R^2^ | 13.685 (.188); 0.053 | 5.683 (.841); 0.094 | 10.422 (.404); 0.064 | **36.924 (<.001); 0.148** | 8.559 (.574); 0.06 |

The values in parentheses for the predictors correspond to the 95% confidence intervals. *Statistically significant association (p < .05).

Categorical variables codification: Binge drinking (less than monthly=0; monthly, weekly, or daily=1); Vaping (no=0; yes=1); Smoking (no=0; yes=1); BMI (BMI<25=0; BMI≥25=1); General health (good, very good, excellent=0; fair, poor=1); Sex (male=0; female=1).

**Supplementary Table 6.** Unstandardized beta coefficients of associations with PSS-10, physical health, mental health and activity limitations measures in linear regressions, using a matched subsample of 100 females and 100 males.

|  | PSS-10 | Physical health | Mental health | Activity limitations |
| --- | --- | --- | --- | --- |
| Discrimination | **1.413*** (0.688) | 0.010 (0.847) | 1.762 (0.987) | 0.899 (0.66) |
| Social support | **-1.961*** (0.517) | -0.568 (0.636) | **-1.962*** (0.742) | **-1.076*** (0.496) |
| Work strain | **3.709*** (0.673) | 0.59 (0.829) | **3.009*** (0.967) | 0.962 (0.646) |
| Caregiver strain | 0.599 (0.720) | 1.61 (0.887) | 1.249 (1.034) | -0.066 (0.691) |
| Emotional intelligence | -1.205 (0.632) | -0.136 (0.778) | -0.74 (0.908) | -0.202 (0.606) |
| Independence | -0.071 (0.531) | -0.087 (0.654) | 1.215 (0.762) | 0.685 (0.509) |
| Risk-taking | -1.090 (0.607) | -0.064 (0.748) | -1.693 (0.872) | -0.555 (0.583) |
| Age | -0.066 (0.041) | -2.642e-4 (0.051) | -0.049 (0.060) | -0.024 (0.04) |
| Education years | 0.099 (0.143) | 0.043 (0.176) | 0.001 (0.205) | -0.057 (0.137) |
| Sex | 1.551 (1.093) | **2.951*** (1.347) | 2.526 (1.57) | -0.063 (1.049) |
| Constant | **27.875*** (2.398) | 2.457 (2.954) | **10.161*** (3.445) | **5.296*** (2.301) |
| F (p); R^2^ | **9.894 (<.001); 0.344** | 1.474 (.152); 0.072 | **6.201 (<.001); 0.247** | **2.14 (.023); 0.102** |

The values in parentheses for the predictors correspond to the standard error. *Statistically significant association (p < .05).

PSS-10: Perceived Stress Scale. Physical/Mental health refer to the number of days with poor physical/mental health during the last 30 days, while Activity limitations refers to the number of days with activity limitations due to poor physical or mental health during the last 30 days.

Categorical variables codification: Sex (male=0; female=1).

**Supplementary Table 7.** Odds ratios of associations with binge drinking, smoking, BMI and general health measures in logistic regressions, using a representative subsample of the population made up of 50 females and 50 males^†^.

|  | Binge drinking | Smoking | BMI | General health |
| --- | --- | --- | --- | --- |
| Discrimination | 1.315 (0.661;2.62) | 0.614 (0.251;1.501) | 1.835 (0.929;3.625) | 1.227  (0.495;3.046) |
| Social support | 1.017 (0.587;1.761) | 0.845 (0.421;1.694) | 0.97 (0.572;1.646) | 0.896  (0.423;1.897) |
| Work strain | **2.462*** (1.112;5.452) | 3.091 (1.033;9.251) | 1.681 (0.805;3.509) | 2.526 (0.801;7.967) |
| Caregiver strain | 0.792 (0.422;1.486) | 0.624 (0.257;1.513) | 1.272 (0.702;2.303) | 1.689 (0.833;3.424) |
| Emotional intelligence | 0.725 (0.371;1.419) | 1.709 (0.727;4.016) | 0.975 (0.507;1.874) | 0.758 (0.311;1.843) |
| Independence | 0.881 (0.534;1.456) | 0.728 (0.412;1.286) | 1.093 (0.667;1.791) | 0.730 (0.356;1.499) |
| Risk-taking | 1.372 (0.736;2.56) | 0.553 (0.237;1.288) | 0.77 (0.405;1.464) | 1.285 (0.51;3.233) |
| Age | **0.956*** (0.916;0.998) | 1.002 (0.953;1.054) | 1.04 (0.999;1.083) | 0.998 (0.94;1.059) |
| Education years | **1.164*** (1.002;1.353) | 1.009 (0.848;1.199) | 1.037 (0.911;1.18) | 1.109 (0.919;1.34) |
| Sex | 0.627 (0.216;1.822) | 0.874 (0.22;3.475) | **0.232*** (0.075;0.718) | 1.017  (0.242;4.28) |
| Constant | 0.278 (0.029;2.644) | 0.127  (0.006;2.536) | 0.141 (0.014;1.396) | **0.019***  (0.001;0.614) |
| χ² (p); McFadden’s R^2^ | 14.511 (.151); 0.112 | 11.533 (.318); 0.131 | 17.319 (.068); 0.13 | 8.408 (.589); 0.109 |

The values in parentheses for the predictors correspond to the 95% confidence intervals. *Statistically significant association (p < .05).

^†^A logistic regression with Vaping could not be performed with this subsample due to only 3 participants out of 100 categorized as users.

Categorical variables codification: Binge drinking (less than monthly=0; monthly, weekly, or daily=1); Smoking (no=0; yes=1); BMI (BMI<25=0; BMI≥25=1); General health (good, very good, excellent=0; fair, poor=1); Sex (male=0; female=1).

**Supplementary Table 8.** Unstandardized beta coefficients of associations with PSS-10, physical health, mental health and activity limitations measures in linear regressions, using a representative subsample of the population made up of 50 females and 50 males.

|  | PSS-10 | Physical health | Mental health | Activity limitations |
| --- | --- | --- | --- | --- |
| Discrimination | 0.931 (0.936) | -1.593 (1.066) | 1.171 (1.405) | -0.188 (0.825) |
| Social support | **-1.949*** (0.76) | -1.071 (0.865) | **-2.804*** (1.141) | **-1.376*** (0.67) |
| Work strain | **3*** (0.997) | 0.59 (1.136) | 1.902 (1.497) | 1.733 (0.879) |
| Caregiver strain | 1.363 (0.873) | **3.017*** (0.995) | 2.144 (1.312) | 0.393 (0.77) |
| Emotional intelligence | -1.543 (0.917) | 0.924 (1.044) | -0.882 (1.377) | 0.353 (0.808) |
| Independence | -0.333 (0.676) | -0.187 (0.77) | 1.301 (1.015) | 0.371 (0.596) |
| Risk-taking | -0.985 (0.864) | -0.631 (0.984) | -2.524 (1.297) | **-1.632*** (0.761) |
| Age | -0.063 (0.055) | 0.048 (0.062) | -0.021 (0.082) | -0.023 (0.048) |
| Education years | -0.105 (0.186) | 0.072 (0.212) | 0.02 (0.28) | 0.117 (0.164) |
| Sex | 0.291 (1.517) | 2.204 (1.729) | 1.533 (2.278) | 0.154 (1.337) |
| Constant | **27.872*** (3.173) | -0.498 (3.615) | 8.52 (4.764) | 1.747 (2.797) |
| F (p); R^2^ | **3.908 (<.001); 0.305** | **2.204 (.024); 0.199** | **2.902 (.003); 0.246** | 1.843 (.064); 0.172 |

The values in parentheses for the predictors correspond to the standard error. *Statistically significant association (p < .05).

PSS-10: Perceived Stress Scale. Physical/Mental health refer to the number of days with poor physical/mental health during the last 30 days, while Activity limitations refers to the number of days with activity limitations due to poor physical or mental health during the last 30 days.

Categorical variables codification: Sex (male=0; female=1).
