# Supplementary material for: Exploring the impact of gender-related variables on health measures and perceived stress
Source: Front Psychol. 2025 Feb 25;16:1500674. doi: 10.3389/fpsyg.2025.1500674 (PMC11893839; doi:10.3389/fpsyg.2025.1500674)
Supplement: Supplementary file 2 [file Data_Sheet_1.pdf]

Dados sociodemográficos

Qual é o seu país de residência?

- ◯ Afeganistão
- ◯ África do Sul
- ◯ Akrotiri
- ◯ Albânia
- ◯ Alemanha
- ◯ Andorra
- ◯ Angola
- ◯ Anguila
- ◯ Antárctida
- ◯ Antígua e Barbuda
- ◯ Arábia Saudita
- ◯ Arctic Ocean
- ◯ Argélia
- ◯ Argentina
- ◯ Arménia
- ◯ Aruba
- ◯ Ashmore and Cartier Islands
- ◯ Atlantic Ocean
- ◯ Austrália
- ◯ Áustria
- ◯ Azerbaijão
- ◯ Baamas
- ◯ Bangladeche
- ◯ Barbados
- ◯ Barém
- ◯ Bélgica
- ◯ Belize
- ◯ Benim
- ◯ Bermudas
- ◯ Bielorrússia
- ◯ Birmânia
- ◯ Bolívia
- ◯ Bósnia e Herzegovina
- ◯ Botsuana
- ◯ Brasil
- ◯ Brunei
- ◯ Bulgária
- ◯ Burquina Faso
- ◯ Burúndi
- ◯ Butão
- ◯ Cabo Verde
- ◯ Camarões
- ◯ Camboja
- ◯ Canadá
- ◯ Catar
- ◯ Cazaquistão
- ◯ Chade
- ◯ Chile
- ◯ China
- ◯ Chipre
- ◯ Clipperton Island
- ◯ Colômbia
- ◯ Comores
- ◯ Congo-Brazzaville
- ◯ Congo-Kinshasa
- ◯ Coral Sea Islands
- ◯ Coreia do Norte
- ◯ Coreia do Sul
- ◯ Costa do Marfim
- ◯ Costa Rica
- ◯ Croácia
- ◯ Cuba
- ◯ Curacao
- ◯ Dhekelia
- ◯ Dinamarca
- ◯ Domínica
- ◯ Egipto
- ◯ Emiratos Árabes Unidos
- ◯ Equador

◯ Eritreia
◯ Eslováquia
◯ Eslovénia
◯ Espanha
◯ Estados Unidos
◯ Estónia
◯ Etiópia
◯ Faroé
◯ Fiji
◯ Filipinas
◯ Finlândia
◯ França
◯ Gabão
◯ Gâmbia
◯ Gana
◯ Gaza Strip
◯ Geórgia
◯ Geórgia do Sul e Sandwich do Sul
◯ Gibraltar
◯ Granada
◯ Grécia
◯ Gronelândia
◯ Guame
◯ Guatemala
◯ Guernsey
◯ Guiana
◯ Guiné
◯ Guiné Equatorial
◯ Guiné-Bissau
◯ Haiti
◯ Honduras
◯ Hong Kong
◯ Hungria
◯ Iémen
◯ Ilha Bouvet
◯ Ilha do Natal
◯ Ilha Norfolk
◯ Ilhas Caimão
◯ Ilhas Cook
◯ Ilhas dos Cocos
◯ Ilhas Falkland
◯ Ilhas Heard e McDonald
◯ Ilhas Marshall
◯ Ilhas Salomão
◯ Ilhas Turcas e Caicos
◯ Ilhas Virgens Americanas
◯ Ilhas Virgens Britânicas
◯ Índia
◯ Indian Ocean
◯ Indonésia
◯ Irão
◯ Iraque
◯ Irlanda
◯ Islândia
◯ Israel
◯ Itália
◯ Jamaica
◯ Jan Mayen
◯ Japão
◯ Jersey
◯ Jibuti
◯ Jordânia
◯ Kosovo
◯ Kuwait
◯ Laos
◯ Lesoto
◯ Letónia
◯ Líbano
◯ Libéria
◯ Líbia
◯ Listenstaine

◯ Lituânia
◯ Luxemburgo
◯ Macau
◯ Macedónia
◯ Madagáscar
◯ Malásia
◯ Malávi
◯ Maldivas
◯ Mali
◯ Malta
◯ Isle of
◯ Marianas do Norte
◯ Marrocos
◯ Maurícia
◯ Mauritânia
◯ México
◯ Micronésia
◯ Moçambique
◯ Moldávia
◯ Mónaco
◯ Mongólia
◯ Monserrate
◯ Montenegro
◯ Mundo
◯ Namíbia
◯ Nauru
◯ Navassa Island
◯ Nepal
◯ Nicarágua
◯ Níger
◯ Nigéria
◯ Niue
◯ Noruega
◯ Nova Caledónia
◯ Nova Zelândia
◯ Omã
◯ Pacific Ocean
◯ Países Baixos
◯ Palau
◯ Panamá
◯ Papua-Nova Guiné
◯ Paquistão
◯ Paracel Islands
◯ Paraguai
◯ Peru
◯ Pitcairn
◯ Polinésia Francesa
◯ Polónia
◯ Porto Rico
◯ Portugal
◯ Quénia
◯ Quirguizistão
◯ Quiribáti
◯ Reino Unido
◯ República Centro-Africana
◯ República Dominicana
◯ Roménia
◯ Ruanda
◯ Rússia
◯ Salvador
◯ Samoa
◯ Samoa Americana
◯ Santa Helena
◯ Santa Lúcia
◯ São Bartolomeu
◯ São Cristóvão e Neves
◯ São Marinho
◯ São Martinho
◯ São Pedro e Miquelon
◯ São Tomé e Príncipe
◯ São Vicente e Granadinas

- ◯ Sara Ocidental
- ◯ Seicheles
- ◯ Senegal
- ◯ Serra Leoa
- ◯ Sérvia
- ◯ Singapura
- ◯ Sint Maarten
- ◯ Síria
- ◯ Somália
- ◯ Southern Ocean
- ◯ Spratly Islands
- ◯ Sri Lanca
- ◯ Suazilândia
- ◯ Sudão
- ◯ Sudão do Sul
- ◯ Suécia
- ◯ Suíça
- ◯ Suriname
- ◯ Svalbard e Jan Mayen
- ◯ Tailândia
- ◯ Taiwan
- ◯ Tajiquistão
- ◯ Tanzânia
- ◯ Território Britânico do Oceano Índico
- ◯ Territórios Austrais Franceses
- ◯ Timor Leste
- ◯ Togo
- ◯ Tokelau
- ◯ Tonga
- ◯ Trindade e Tobago
- ◯ Tunísia
- ◯ Turquemenistão
- ◯ Turquia
- ◯ Tuvalu
- ◯ Ucrânia
- ◯ Uganda
- ◯ Uruguai
- ◯ Usbequistão
- ◯ Vanuatu
- ◯ Vaticano
- ◯ Venezuela
- ◯ Vietname
- ◯ Wake Island
- ◯ Wallis e Futuna
- ◯ West Bank
- ◯ Zâmbia
- ◯ Zimbabué

Onde nasceu?

- ◯ Afeganistão
- ◯ África do Sul
- ◯ Akrotiri
- ◯ Albânia
- ◯ Alemanha
- ◯ Andorra
- ◯ Angola
- ◯ Anguila
- ◯ Antárctida
- ◯ Antígua e Barbuda
- ◯ Arábia Saudita
- ◯ Arctic Ocean
- ◯ Argélia
- ◯ Argentina
- ◯ Arménia
- ◯ Aruba
- ◯ Ashmore and Cartier Islands
- ◯ Atlantic Ocean
- ◯ Austrália
- ◯ Áustria
- ◯ Azerbaijão
- ◯ Baamas
- ◯ Bangladeche
- ◯ Barbados
- ◯ Barém
- ◯ Bélgica
- ◯ Belize
- ◯ Benim
- ◯ Bermudas
- ◯ Bielorrússia
- ◯ Birmânia
- ◯ Bolívia
- ◯ Bósnia e Herzegovina
- ◯ Botsuana
- ◯ Brasil
- ◯ Brunei
- ◯ Bulgária
- ◯ Burquina Faso
- ◯ Burúndi
- ◯ Butão
- ◯ Cabo Verde
- ◯ Camarões
- ◯ Camboja
- ◯ Canadá
- ◯ Catar
- ◯ Cazaquistão
- ◯ Chade
- ◯ Chile
- ◯ China
- ◯ Chipre
- ◯ Clipperton Island
- ◯ Colômbia
- ◯ Comores
- ◯ Congo-Brazzaville
- ◯ Congo-Kinshasa
- ◯ Coral Sea Islands
- ◯ Coreia do Norte
- ◯ Coreia do Sul
- ◯ Costa do Marfim
- ◯ Costa Rica
- ◯ Croácia
- ◯ Cuba
- ◯ Curacao
- ◯ Dhekelia
- ◯ Dinamarca
- ◯ Domínica
- ◯ Egipto
- ◯ Emiratos Árabes Unidos
- ◯ Equador

◯ Eritreia
◯ Eslováquia
◯ Eslovénia
◯ Espanha
◯ Estados Unidos
◯ Estónia
◯ Etiópia
◯ Faroé
◯ Fiji
◯ Filipinas
◯ Finlândia
◯ França
◯ Gabão
◯ Gâmbia
◯ Gana
◯ Gaza Strip
◯ Geórgia
◯ Geórgia do Sul e Sandwich do Sul
◯ Gibraltar
◯ Granada
◯ Grécia
◯ Gronelândia
◯ Guame
◯ Guatemala
◯ Guernsey
◯ Guiana
◯ Guiné
◯ Guiné Equatorial
◯ Guiné-Bissau
◯ Haiti
◯ Honduras
◯ Hong Kong
◯ Hungria
◯ Iémen
◯ Ilha Bouvet
◯ Ilha do Natal
◯ Ilha Norfolk
◯ Ilhas Caimão
◯ Ilhas Cook
◯ Ilhas dos Cocos
◯ Ilhas Falkland
◯ Ilhas Heard e McDonald
◯ Ilhas Marshall
◯ Ilhas Salomão
◯ Ilhas Turcas e Caicos
◯ Ilhas Virgens Americanas
◯ Ilhas Virgens Britânicas
◯ Índia
◯ Indian Ocean
◯ Indonésia
◯ Irão
◯ Iraque
◯ Irlanda
◯ Islândia
◯ Israel
◯ Itália
◯ Jamaica
◯ Jan Mayen
◯ Japão
◯ Jersey
◯ Jibuti
◯ Jordânia
◯ Kosovo
◯ Kuwait
◯ Laos
◯ Lesoto
◯ Letónia
◯ Líbano
◯ Libéria
◯ Líbia
◯ Listenstaine

◯ Lituânia
◯ Luxemburgo
◯ Macau
◯ Macedónia
◯ Madagáscar
◯ Malásia
◯ Malávi
◯ Maldivas
◯ Mali
◯ Malta
◯ Isle of
◯ Marianas do Norte
◯ Marrocos
◯ Maurícia
◯ Mauritânia
◯ México
◯ Micronésia
◯ Moçambique
◯ Moldávia
◯ Mónaco
◯ Mongólia
◯ Monserrate
◯ Montenegro
◯ Mundo
◯ Namíbia
◯ Nauru
◯ Navassa Island
◯ Nepal
◯ Nicarágua
◯ Níger
◯ Nigéria
◯ Niue
◯ Noruega
◯ Nova Caledónia
◯ Nova Zelândia
◯ Omã
◯ Pacific Ocean
◯ Países Baixos
◯ Palau
◯ Panamá
◯ Papua-Nova Guiné
◯ Paquistão
◯ Paracel Islands
◯ Paraguai
◯ Peru
◯ Pitcairn
◯ Polinésia Francesa
◯ Polónia
◯ Porto Rico
◯ Portugal
◯ Quénia
◯ Quirguizistão
◯ Quiribáti
◯ Reino Unido
◯ República Centro-Africana
◯ República Dominicana
◯ Roménia
◯ Ruanda
◯ Rússia
◯ Salvador
◯ Samoa
◯ Samoa Americana
◯ Santa Helena
◯ Santa Lúcia
◯ São Bartolomeu
◯ São Cristóvão e Neves
◯ São Marinho
◯ São Martinho
◯ São Pedro e Miquelon
◯ São Tomé e Príncipe
◯ São Vicente e Granadinas

◯ Sara Ocidental
◯ Seicheles
◯ Senegal
◯ Serra Leoa
◯ Sérvia
◯ Singapura
◯ Sint Maarten
◯ Síria
◯ Somália
◯ Southern Ocean
◯ Spratly Islands
◯ Sri Lanca
◯ Suazilândia
◯ Sudão
◯ Sudão do Sul
◯ Suécia
◯ Suíça
◯ Suriname
◯ Svalbard e Jan Mayen
◯ Tailândia
◯ Taiwan
◯ Tajiquistão
◯ Tanzânia
◯ Território Britânico do Oceano Índico
◯ Territórios Austrais Franceses
◯ Timor Leste
◯ Togo
◯ Tokelau
◯ Tonga
◯ Trindade e Tobago
◯ Tunísia
◯ Turquemenistão
◯ Turquia
◯ Tuvalu
◯ Ucrânia
◯ Uganda
◯ Uruguai
◯ Usbequistão
◯ Vanuatu
◯ Vaticano
◯ Venezuela
◯ Vietname
◯ Wake Island
◯ Wallis e Futuna
◯ West Bank
◯ Zâmbia
◯ Zimbabué

Há quanto tempo reside no país onde se encontra atualmente?

____
(Indicar tempo em anos)

Em que ano nasceu?

____

Número total de anos de escolaridade

____

Nível de escolaridade mais elevado	◯ Nenhum<br>◯ Ensino Básico - 1º Ciclo<br>◯ Ensino Básico - 2º Ciclo<br>◯ Ensino Básico - 3º Ciclo<br>◯ Ensino Secundário<br>◯ Licenciatura<br>◯ Mestrado<br>◯ Doutoramento
Qual foi o seu rendimento anual médio no ano passado?	◯ Menos de 7000€<br>◯ 7000€ - 10999€<br>◯ 11000€ - 19999€<br>◯ 20000€ - 24999€<br>◯ 25000€ - 36999€<br>◯ 37000€ - 79999€<br>◯ Mais do que 80000€<br>◯ Prefiro não responder<br>(Por favor, considere todos os seus rendimentos, incluindo salário, recibos verdes, rendimentos/lucros/ganhos provenientes de pequenas empresas, segurança social, pagamentos das forças armadas, bónus em dinheiro e ajudas de custo)
Estado civil	◯ Solteiro(a)<br>◯ Casado(a)/União de facto<br>◯ Divorciado(a)/Separado(a)<br>◯ Viúvo(a)
Qual foi o seu sexo à nascença?	◯ Masculino<br>◯ Feminino<br>◯ Intersexual<br>◯ Prefiro não responder
Qual é o seu género?	☐ Homem<br>☐ Mulher<br>☐ Fluído/Não binário<br>☐ Outro<br>☐ Prefiro não responder<br>(Por favor, selecione todas as respostas que se aplicam)
Por favor, especifique:	______________________________
Foi criado como:	◯ Rapaz<br>◯ Rapariga<br>◯ Outro<br>◯ Prefiro não responder
Por favor, especifique:	______________________________
Considera-se trans? (ex: transgénero, transsexual)	◯ Sim<br>◯ Não<br>◯ Prefiro não responder

Como descreveria a sua orientação sexual?	◯ Exclusivamente heterossexual<br>◯ Maioritariamente heterossexual<br>◯ Bissexual<br>◯ Maioritariamente gay/lésbica<br>◯ Exclusivamente gay/lésbica<br>◯ Pansexual<br>◯ Assexual<br>◯ Outra<br>◯ Prefiro não responder
Por favor, especifique:	__________________________________
Qual é a sua religião?	◯ Sem religião (Ex: ateu, agnóstico)<br>◯ Católico(a)<br>◯ Outra<br>◯ Prefiro não responder
Por favor, especifique:	__________________________________
Considera-se uma pessoa:	◯ Nada religiosa<br>◯ Pouco religiosa<br>◯ Moderadamente religiosa<br>◯ Bastante religiosa<br>◯ Muito religiosa
É praticante?	◯ Sim<br>◯ Não<br>◯ Não se aplica

Dados de saúde

Em geral, como diria que é a sua saúde:	◯ Excelente<br>◯ Muito boa<br>◯ Boa<br>◯ Razoável<br>◯ Fraca
Agora pense na sua saúde física, que inclui doenças físicas e lesões, quantos dias dos últimos 30 dias a sua saúde física não foi boa?	______________________
Agora pense na sua saúde mental, que inclui stress, depressão e problemas emocionais, quantos dias dos últimos 30 dias a sua saúde mental não foi boa?	______________________
Durante os últimos 30 dias, aproximadamente quantos dias a sua saúde física ou mental fraca o(a) impediu de realizar as suas atividades habituais, como cuidados pessoais, trabalho, ou lazer?	______________________
Quantos cigarros fuma por dia, excluindo os cigarros eletrónicos?	______________________
Quantas vezes faz vaping ou utiliza cigarros eletrónicos por dia?	______________________
Nos últimos 3 meses, com que frequência consumiu 5 ou mais bebidas alcoólicas numa ocasião?	◯ Nunca<br>◯ Menos de uma vez por mês<br>◯ Mensalmente<br>◯ Semanalmente<br>◯ Diariamente ou quase diariamente
Nos últimos 3 meses, com que frequência consumiu 4 ou mais bebidas alcoólicas numa ocasião?	◯ Nunca<br>◯ Menos de uma vez por mês<br>◯ Mensalmente<br>◯ Semanalmente<br>◯ Diariamente ou quase diariamente
Altura	______________________<br>(cm)
Peso	______________________<br>(kg)
