# Supplementary material for: Exploring the impact of gender-related variables on health measures and perceived stress
Source: Front Psychol. 2025 Feb 25;16:1500674. doi: 10.3389/fpsyg.2025.1500674 (PMC11893839; doi:10.3389/fpsyg.2025.1500674)
Supplement: Supplementary file 3 [file Data_Sheet_2.pdf]

Questionário de Stanford de Variáveis relacionadas com o Género para a Investigação em Saúde (GVHR)

Nielsen et al. (2021)

Versão portuguesa de Picó-Pérez et al. (2025)

Situação profissional	◯ Trabalhador(a)<br>◯ Estudante<br>◯ Desempregado(a)<br>◯ Reformado(a)<br>◯ Doença permanente/Incapacidade<br>◯ Doméstico(a) ou a cuidar da família<br>(Escolha a opção que melhor represente a sua situação atual)

As pessoas vêem-se de formas diferentes. Não há respostas certas ou erradas às perguntas que se seguem. Apenas queremos saber aquilo que é verdade para si. Escolha a resposta que melhor o(a) descreve.

Em geral, quão preparado(a) está para correr riscos?	◯ Nada preparado(a)<br>◯ Ligeiramente preparado(a)<br>◯ Moderadamente preparado(a)<br>◯ Muito preparado(a)<br>◯ Completamente preparado(a)
Quão preparado(a) está para correr riscos ao tomar decisões financeiras?	◯ Nada preparado(a)<br>◯ Ligeiramente preparado(a)<br>◯ Moderadamente preparado(a)<br>◯ Muito preparado(a)<br>◯ Completamente preparado(a)
Quão preparado(a) está para correr riscos no que toca a atividades recreativas?	◯ Nada preparado(a)<br>◯ Ligeiramente preparado(a)<br>◯ Moderadamente preparado(a)<br>◯ Muito preparado(a)<br>◯ Completamente preparado(a)
Quão importante é para si ser independente?	◯ Extremamente importante<br>◯ Muito importante<br>◯ Moderadamente importante<br>◯ Ligeiramente importante<br>◯ Nada importante
Quão importante é para si resolver os seus problemas sozinho(a)?	◯ Extremamente importante<br>◯ Muito importante<br>◯ Moderadamente importante<br>◯ Ligeiramente importante<br>◯ Nada importante

Citation: *Picó-Pérez M, Coelho MS, Vieira R, Machado-Sousa M and Morgado P (2025). Exploring the impact of gender-related variables on health measures and perceived stress. Front. Psychol. 16:1500674. doi: 10.3389/fpsyg.2025.1500674*

As pessoas vêem-se de formas diferentes. Não há respostas certas ou erradas às perguntas que se seguem. Apenas queremos saber aquilo que é verdade para si. Escolha a resposta que melhor o(a) descreve.	
Com que frequência os(as) seus(suas) amigos(as) falam consigo sobre os problemas deles(as)?	◯ Nunca<br>◯ Raramente<br>◯ Às vezes<br>◯ Quase sempre<br>◯ Sempre
Com que frequência fala com os(as) seus(suas) amigos(as) sobre os seus problemas?	◯ Nunca<br>◯ Raramente<br>◯ Às vezes<br>◯ Quase sempre<br>◯ Sempre
Quão fácil é para si expressar o que está a sentir aos outros?	◯ Nada fácil<br>◯ Ligeiramente fácil<br>◯ Moderadamente fácil<br>◯ Muito fácil<br>◯ Extremamente fácil
Atualmente é responsável por cuidar de alguém?	◯ Atualmente sou responsável por cuidar de alguém<br>◯ Fui responsável por cuidar de alguém no passado<br>◯ Nunca fui responsável por cuidar de alguém<br>◯ Atualmente sou responsável por cuidar de alguém e fui responsável por cuidar de alguém no passado<br>(Com "cuidar de alguém", queremos dizer prestar assistência e apoio não remunerados a alguém com necessidades físicas ou psicológicas, como uma criança, um(a) idoso(a), um(a) parceiro(a) ou um(a) familiar com deficiência)

Se na pergunta anterior respondeu "Nunca fui responsável por cuidar de alguém", pode ignorar as 3 perguntas seguintes.

No ano passado, com que frequência se sentiu emocionalmente exausto(a) por causa das suas responsabilidades de cuidador(a)?	◯ Nunca<br>◯ Raramente<br>◯ Às vezes<br>◯ Quase sempre<br>◯ Sempre
No ano passado, com que frequência se sentiu fisicamente exausto(a) por causa das suas responsabilidades de cuidador(a)?	◯ Nunca<br>◯ Raramente<br>◯ Às vezes<br>◯ Quase sempre<br>◯ Sempre
No ano passado, com que frequência as suas responsabilidades de cuidador(a) fizeram-no(a) preocupar-se sobre o futuro?	◯ Nunca<br>◯ Raramente<br>◯ Às vezes<br>◯ Quase sempre<br>◯ Sempre

Citation: *Picó-Pérez M, Coelho MS, Vieira R, Machado-Sousa M and Morgado P (2025). Exploring the impact of gender-related variables on health measures and perceived stress. Front. Psychol. 16:1500674. doi: 10.3389/fpsyg.2025.1500674*

**Estamos interessados(as) em saber como passa o seu tempo num dia da semana normal, de segunda a sexta-feira. Por favor indique a sua melhor estimativa. Em média, quantas horas por dia passa no seguinte:**<br>**O total não deve exceder 24 horas.**

Trabalhar (trabalho remunerado, estudar, estágios, etc.):	______________________________
Cuidar de alguém (cuidar de crianças, idosos(as), parceiros(as), etc.):	______________________________

Responda às seguintes perguntas desta página apenas se a sua situação profissional atual é Trabalhador(a) e/ou Estudante.

Estamos interessados(as) em saber como se sente em relação ao seu emprego atual, incluindo as suas atividades diárias de trabalho como funcionário(a) ou estudante. Para cada uma das perguntas a seguir, selecione a resposta que melhor descreve as suas atividades de trabalho. Se tem vários empregos, por favor pense no emprego em que passa mais horas por semana.

Com que frequência o seu emprego exige trabalhar a um ritmo acelerado?	◯ Nunca<br>◯ Raramente<br>◯ Às vezes<br>◯ Quase sempre<br>◯ Sempre
Com que frequência o seu emprego envolve tarefas repetitivas?	◯ Nunca<br>◯ Raramente<br>◯ Às vezes<br>◯ Quase sempre<br>◯ Sempre
Com que frequência se sente emocionalmente exausto(a) das suas atividades de trabalho?	◯ Nunca<br>◯ Raramente<br>◯ Às vezes<br>◯ Quase sempre<br>◯ Sempre
Com que frequência se sente fisicamente exausto(a) das suas atividades de trabalho?	◯ Nunca<br>◯ Raramente<br>◯ Às vezes<br>◯ Quase sempre<br>◯ Sempre

Citation: *Picó-Pérez M, Coelho MS, Vieira R, Machado-Sousa M and Morgado P (2025). Exploring the impact of gender-related variables on health measures and perceived stress. Front. Psychol. 16:1500674. doi: 10.3389/fpsyg.2025.1500674*

Às vezes, as pessoas procuram nos outros companheirismo, ajuda, ou outros tipos de suporte físico ou emocional. As próximas perguntas questionam sobre o suporte disponível quando precisar dele. Escolha a resposta que melhor descreve a sua situação.

No ano passado, com que frequência teve alguém para lhe demonstrar amor e carinho?

- ◯ Nunca
- ◯ Raramente
- ◯ Às vezes
- ◯ Quase sempre
- ◯ Sempre

No ano passado, com que frequência teve alguém para ajudá-lo(a) com as tarefas diárias?

- ◯ Nunca
- ◯ Raramente
- ◯ Às vezes
- ◯ Quase sempre
- ◯ Sempre

As próximas perguntas questionam a frequência com que se sentiu discriminado(a) por causa do seu género. Pode não ter certeza das respostas a estas perguntas, mas gostaríamos que escolhesse a resposta que melhor descreve a sua experiência.

Por causa do seu género, com que frequência se sentiu discriminado(a)?

- ◯ Nunca
- ◯ Raramente
- ◯ Às vezes
- ◯ Quase sempre
- ◯ Sempre

Por causa do seu género, com que frequência se sentiu discriminado(a)...

	Nunca	Raramente	Às vezes	Quase sempre	Sempre
Ao ser contratado(a)	◯	◯	◯	◯	◯
Na escola	◯	◯	◯	◯	◯
Ao receber cuidados médicos	◯	◯	◯	◯	◯
Em contextos públicos	◯	◯	◯	◯	◯
Na sua família	◯	◯	◯	◯	◯

Citation: *Picó-Pérez M, Coelho MS, Vieira R, Machado-Sousa M and Morgado P (2025). Exploring the impact of gender-related variables on health measures and perceived stress. Front. Psychol. 16:1500674. doi: 10.3389/fpsyg.2025.1500674*
